# Anisotropically Wettable Porous Transport Layers for Gas Management in Water Electrolyzers

**DOI:** 10.1002/advs.202508569

**Published:** 2025-11-08

**Authors:** Yunseok Kang, Seunghyun Lee, Jinseo Lee, Soi Lee, Geonwoo Lee, Hyeongoo Kim, Gwan Hyun Choi, Jungki Ryu, Dong Woog Lee

**Affiliations:** ^1^ School of Energy and Chemical Engineering Ulsan National Institute of Science and Technology (UNIST) Ulsan 44919 Republic of Korea; ^2^ Emergent Hydrogen Technology R&D Center Ulsan National Institute of Science and Technology (UNIST) Ulsan 44919 Republic of Korea; ^3^ Graduate School of Carbon Neutrality Ulsan National Institute of Science and Technology (UNIST) Ulsan 44919 Republic of Korea; ^4^ Center for Renewable Carbon Ulsan National Institute of Science and Technology (UNIST) Ulsan 44919 Republic of Korea; ^5^ Department of Chemistry University of California Berkeley CA 94720 USA

**Keywords:** electrolyzers, hydrogen, mass transport, porous transport layers, water electrolysis

## Abstract

Conventional studies on water electrolysis have primarily focused on designing novel electrocatalysts and membranes, with intrinsic properties closely linked to the immediate performance of water electrolyzers. However, less attention is directed toward porous transport layers (PTLs), which are essential for sustaining efficient, long‐term, high‐current operation by enabling effective mass transport. Here, a novel PTL with anisotropic wettability (AW‐PTL) is introduced to enhance the efficiency of anion exchange membrane water electrolyzers (AEMWEs). By hydrophobically modifying the upper half of hydrophilic Ni foam with polytetrafluoroethylene using a simple spray‐coating method, anisotropic wettability is achieved, enabling the directional transport of liquid electrolytes and gaseous products. This design significantly improves AEMWE efficiency by facilitating the removal of gas bubbles, which typically block catalyst active sites and hinder electrolyte supply. The method is universally applicable across conventional PTL types and demonstrates scalability to large‐area (up to 225 cm^2^) and short‐stack AEMWEs. This work advances the practical application of water electrolysis, providing an adaptable solution for other water electrolyzer types using existing catalysts and membranes.

## Graphical abstract

Synergistic wetting, achieved by integrating hydrophilic and hydrophobic domains, enhances mass transport in porous transport layers. In situ visualization reveals that dual‐phase wetting suppresses gas accumulation and promotes continuous bubble release, enabling stable, bubble‐free electrolysis within membrane electrode assemblies. This simple yet powerful strategy overcomes transport limitations and advances porous transport layer design for efficient gas/liquid diffusion.

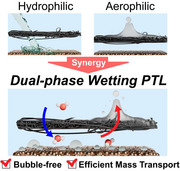

## 1 Introduction

The efficient, clean, and large‐scale production of hydrogen through water electrolysis using renewable electricity is one of the most urgent yet challenging tasks in addressing current environmental issues. Given hydrogen's versatile role in decarbonizing various sectors, such as electricity generation,^[^ ^1^ ^]^ chemical production,^[^ ^2^, ^3^ ^]^ and transportation,^[^ ^4^, ^5^ ^]^ its clean production with minimal CO_2_ emissions is increasingly essential. For example, intermittent renewable electricity can be effectively stored and utilized through water electrolyzers^[^ ^6^ ^]^ and fuel cells.^[^ ^7^ ^]^ Additionally, hydrogenation is a key step in the carbon‐neutral production of conventional liquid fuels and chemicals from recyclable carbon resources, such as biomass^[^ ^8^ ^]^ and plastic wastes.^[^ ^9^ ^]^ Consequently, hydrogen demand is expected to grow significantly from its current scale of 90 million tons.^[^ ^10^ ^]^

In this regard, substantial attention has been directed toward developing efficient water electrolyzers and their functional components. Various forms of water electrolyzers have been developed, ranging from conventional alkaline water electrolyzers (AWEs)^[^ ^11^, ^12^ ^]^ to next‐generation technologies like proton exchange membrane water electrolyzers (PEMWEs),^[^ ^13^, ^14^, ^15^ ^]^ anion exchange membrane water electrolyzers (AEMWEs),^[^ ^16^ ^]^ and bipolar membrane water electrolyzers (BPMWEs).^[^ ^17^ ^]^ Accordingly, numerous studies have focused on designing novel electrocatalysts to reduce overpotentials for hydrogen evolution reactions (HER)^[^ ^18^, ^19^, ^20^ ^]^ and oxygen evolution reactions (OER)^[^ ^21^, ^22^ ^]^ under both acidic^[^ ^18^, ^21^ ^]^ and basic^[^ ^19^, ^20^, ^22^ ^]^ conditions. Additionally, efforts have been made to develop advanced membranes^[^ ^23^, ^24^, ^25^ ^]^ that enable selective ion migration, improve ion conductivity, and suppress gas crossover.

However, the performance of electrocatalysts and membranes in practical water electrolyzers—especially during long‐term, high‐current operations—often falls short of expectations,^[^ ^26^, ^27^, ^28^ ^]^ primarily due to mass transport issues. Moreover, the unique nature of water electrolysis, which generates a dynamic three‐phase interface involving solid electrodes, liquid electrolytes, and gaseous products,^[^ ^29^ ^]^ further complicates efficient mass transport.^[^ ^30^, ^31^, ^32^, ^33^ ^]^ This results in performance degradation and discrepancies between the intrinsic properties of electrocatalysts and membranes and their actual performance under practical high‐current conditions, largely due to increased ohmic and mass transport overpotentials.

In this context, designing effective porous transport layers (PTLs) is both academically and practically essential, as they provide pathways for electrolyte supply and gas removal from the catalyst surface. Recent studies have underscored the critical role of PTLs, their limitations, and emerging strategies for controlling wettability and structure.^[^ ^34^, ^35^ ^]^ For example, approaches such as introducing hydrophobic domains and patterning have been explored.^[^ ^36^, ^37^ ^]^ However, these methods often rely on complex and costly fabrication processes and have been demonstrated only in relatively small‐area electrolyzers. Furthermore, PTLs remain insufficiently understood with respect to their real‐time bubble transport dynamics in operating electrolyzers and their broader applicability across diverse catalysts (Table S1, Supporting Information).

In this study, we report a novel PTL with anisotropic wettability (AW‐PTL) for efficient AEMWEs. AW‐PTLs were readily prepared by modifying the upper half of conventional PTLs with polytetrafluoroethylene (PTFE). While the hydrophilic pristine Ni foam (NF), commonly used as a PTL in alkaline conditions, readily blocked gas bubble transport, NF‐based AW‐PTLs facilitated the transport of gas bubbles from the hydrophilic to the hydrophobic PTFE‐modified side due to a significant reduction in the bubble point pressure (BPP). Overpotential breakdown analysis indicated a substantial reduction in mass transport overpotential, especially at higher current densities, when using AW‐PTLs, while ohmic and kinetic overpotentials remained similar regardless of the PTL type. Real‐time observation using a custom‐made transparent cell demonstrated continuous, efficient gas bubble removal without trapping within the electrolyzer. Due to the high chemical stability of PTFE, AW‐PTLs remained stable for at least 150 h, even at 500 mA cm^−2^. Since AW‐PTLs were prepared using simple spray‐coating methods, our approach is readily scalable in terms of electrolyzer area and stack number and is generally applicable to various PTL types, regardless of the catalysts used. This study provides valuable insights into the design and practical application of water electrolyzers for a sustainable future.

## 2 Results

### 2.1 Preparation of AW‐PTLs

**Figure 1:**
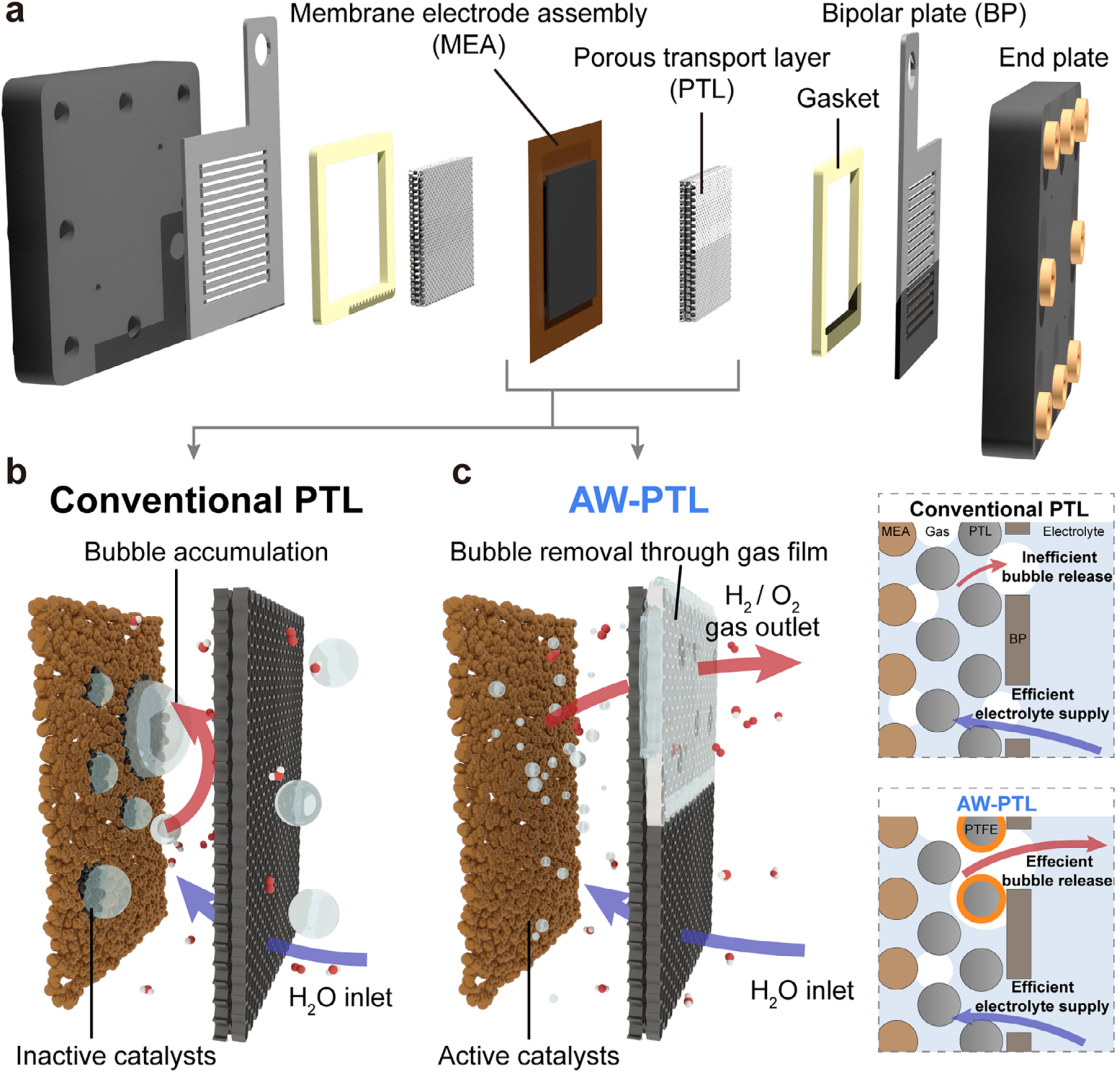
Components of water electrolyzers and the role of PTLs. a) Graphical illustration showing the typical components of AEMWEs. Gas bubble dynamics in b) conventional PTLs, showing bubble accumulation, and c) AW‐PTLs, where partial PTFE modification enables efficient bubble removal through a gas film. The accompanying 2D schematics illustrate how uncoated hydrophilic domains secure electrolyte supply and electronic conduction, while PTFE‐coated domains provide preferential pathways for gas release. In the device architecture, the Ni foam serves as PTL beneath the catalyst layer (e.g., NiFe for OER and Pt/C for HER). The PTFE coating was applied only to the PTL region, leaving the catalyst layer uncoated.

PTLs play a critical role in providing electrical contact to electrocatalysts and creating pathways for electrolyte supply and product removal,^[^ ^33^, ^38^, ^39^ ^]^ regardless of the type of water electrolyzers, such as AEMWEs (**Figure** **1**a), PEMWEs, and BPMWEs. However, most conventional PTLs are highly hydrophilic, which causes hydrogen and oxygen gas bubbles generated during electrolysis to become trapped between the catalyst layers and PTLs.^[^ ^40^ ^]^ This results in active site blocking and significant performance degradation, even with state‐of‐the‐art catalysts and membranes (Figure 1b).^[^ ^41^ ^]^

To address this issue, we modified one side of the hydrophilic PTLs with hydrophobic PTFEs using a simple spray‐coating method. PTFE was selected due to its aerophilicity and high chemical stability; for example, it remained stable in 1 m KOH at 80 °C for 20 days (Figure S1, Supporting Information). Although NFs were primarily used as model PTLs for AEMWEs in this study, other types of PTLs, such as Ni fiber felts and stainless steel (SUS) foam, can also be employed when necessary. The entire upper side of the PTLs was coated with PTFE for surface characterizations, while only the upper half of the PTLs was modified for electrolyzer applications. We hypothesized that such PTLs could significantly improve electrolyzer performance, as the modified section would allow for the rapid escape of gas bubbles without trapping, while the unmodified section would ensure proper electrical contact and electrolyte supply (Figure S2, Supporting Information).

**Figure 2:**
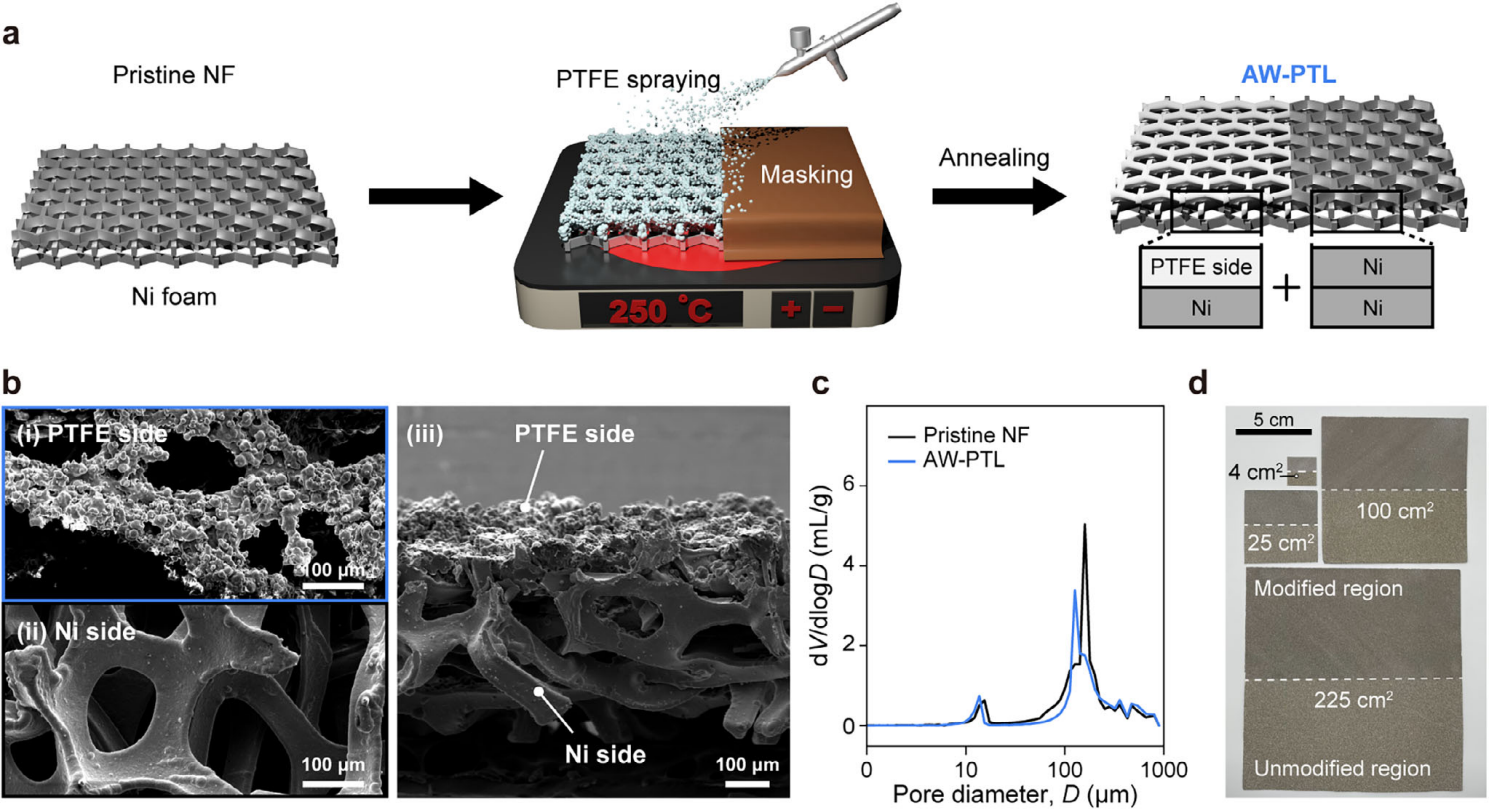
Fabrication and characterization of AW‐PTLs. a) Fabrication process of AW‐PTLs by spray‐coating and annealing. b) Electron micrographs of NF‐based AW‐PTLs: i) the PTFE‐coated side, ii) the Ni side, and iii) the cross‐section of AW‐PTLs. c) Pore size distribution of pristine NF and AW‐PTLs. d) Photographs demonstrating the scalability of our approach.

The coating process was conducted in two steps: spraying followed by annealing (**Figure** **2**a). During the spraying process, NFs with a thickness of 0.5 mm were heated to 250 °C to minimize the permeation of the PTFE dispersion into the backside of the NF (Figures S3 and S4, Supporting Information). Subsequent annealing at 370 °C caused the agglomeration of PTFE particles and mechanical interlocking between the PTFE and NF, resulting in a single‐sided PTFE coating with a slight reduction in pore size (Figure 2b,c) without PTFE degradation (Figures S5 and S6, Supporting Information). Furthermore, through‐plane conductivity measurements confirmed that complete PTFE coverage caused a significant decrease in conductivity, whereas half PTFE coverage maintained values comparable to pristine NF, indicating that partial modification does not compromise electron transport (Figure S7, Supporting Information). Due to its simplicity, our spray‐coating method can be generally applied to various types of PTLs, including thicker NFs (1.6 mm thick), Ni fiber felt, and dense SUS foam with smaller pores, though slight adjustments to the preparation conditions may be necessary (Figure S8, Supporting Information). Given the nature of the spray‐coating method, AW‐PTLs with a larger area (225 cm^2^) were easily and uniformly fabricated (Figure 2d; Figure S9, Supporting Information).

### 2.2 Wetting Properties of AW‐PTLs

The wetting properties of NF‐based AW‐PTLs were evaluated by measuring static and dynamic contact angles (Figure S10 and Table S2, Supporting Information). NF‐based AW‐PTLs exhibited anisotropic wettability; the PTFE‐modified side displayed superhydrophobicity, while the unmodified Ni side was extremely hydrophilic, readily absorbing water droplets like pristine NF (Figure S10a, Supporting Information).^[^ ^42^ ^]^ When measuring air bubble contact angles underwater, pristine NF showed high aerophobicity with a contact angle of 146°. In contrast, AW‐PTLs exhibited superaerophilicity on both surfaces (Figure S10b, Supporting Information). This behavior of AW‐PTLs can be attributed to experimental conditions where the NFs were positioned near the air‐water interface, with air trapped in the thicker PTFE‐coated layer. During dynamic contact angle measurements with substrate withdrawal from water, pristine NFs displayed symmetric capillary rise, whereas AW‐PTLs exhibited asymmetric capillary effects (Figure S10c, Supporting Information), demonstrating the anisotropic wettability of the AW‐PTLs.

Based on these results, water permeability tests were conducted in air (Figure S11, Supporting Information). The pristine NF fully absorbed water droplets due to its hydrophilic nature. For dry NF‐based AW‐PTLs, the bare Ni‐exposed side allowed good wetting of a water droplet, while the PTFE‐coated side repelled it. However, once the bare Ni side was wetted, a water droplet penetrated through it within 148 ms—much longer than the 5 ms observed for pristine NF (Figure S12, Supporting Information).

**Figure 3:**
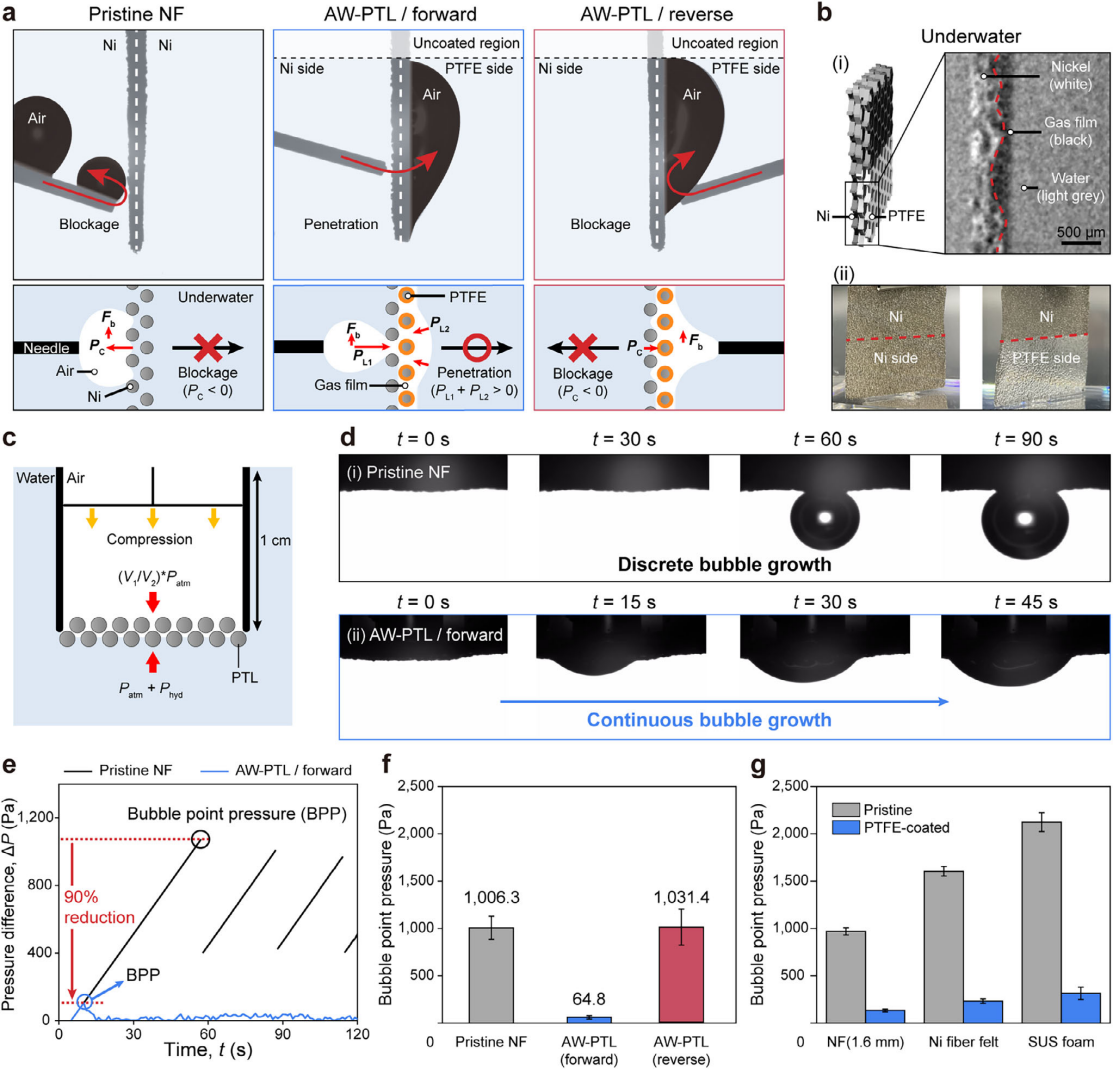
Gas permeation properties of AW‐PTL. a) Gas permeation properties of pristine NF and AW‐PTL: (first row) photographs taken during permeation tests and (second row) schematic illustrations explaining the respective permeation mechanisms. *P*_C_, *P*_L1_, *P*_L2,_ and *F*_b_ denote capillary pressure, Laplace pressure of a bubble, Laplace pressure of a gas film, and buoyancy of a bubble, respectively. b) Formation of the gas film on AW‐PTLs: i) Cross‐sectional X‐ray computed tomography of the water‐immersed AW‐PTL and ii) photographs of pristine NF and AW‐PTL upon immersion in water. c) Schematic representation of the bubble point pressure (BPP) measurement setup, where *V*_1_ and *V*_2_ represent the initial and compressed volume of the syringe, and *P*_atm_ and *P*_hyd_ represent atmospheric and hydrostatic pressure, respectively. d) Bubble penetration behavior for i) pristine NF and ii) AW‐PTL in the forward direction. e) Pressure profiles and f) BPPs of pristine NF and AW‐PTL, and g) of various PTLs before and after the modification.

Next, we investigated the gas permeability of pristine and modified NFs underwater (**Figure** **3**a). As expected, pristine NFs repelled gas bubbles due to their high capillary pressure. In contrast, AW‐PTLs exhibited anisotropic gas bubble wetting and permeability behaviors, distinct from their water wetting behaviors. Gas bubbles readily penetrated through the AW‐PTL in the forward direction—from the hydrophilic bare Ni side to the hydrophobic PTFE‐coated side—but not in the reverse direction (Figure 3a; Figure S13, and Movies S1 and S2). On the hydrophilic side, bubble penetration is inhibited; however, external forces (e.g., gas pressure or buoyancy) can drive the bubble into contact with the adjacent air film, where subsequent coalescence enables unidirectional transport toward the PTFE side.^[^ ^43^ ^]^ We hypothesized that a gas film trapped on the PTFE‐coated side upon immersion (i.e., Cassie–Baxter state) was responsible for the observed anisotropic permeation behavior. The presence of a gas film within the AW‐PTLs was confirmed by X‐ray computed tomography and was even visible to the naked eye (Figure 3b). When fully immersing AW‐PTLs for AEMWEs (i.e., NFs with partial modification on one side) in water, the PTFE side formed a gas film due to its superaerophilicity, while the bare Ni side was fully wetted (i.e., Wenzel state) (Figure 3b; Figure S10c, Supporting Information). To confirm the effect of the gas film on the anisotropic gas permeation properties, we tested gas permeation after degassing; no gas penetration was observed, even in the forward direction (Figure S14, Supporting Information). Theoretically, the role of the gas film can be explained as follows. The capillary pressure^[^ ^44^ ^]^ of the hydrophilic Ni side repels gas bubbles until they contact the gas film. Once the bubble encounters the gas film, a gas channel forms, enabling bubble penetration. The Laplace pressure^[^ ^45^ ^]^ difference induces spontaneous unidirectional penetration from the bubbles (high pressure) to the gas film (low pressure).

We experimentally investigated the bubble penetration mechanism by observing real‐time penetration behavior and measuring bubble point pressure (BPP) (Figure 3c,d; Movies S3 and S4, Supporting Information). While pristine NFs exhibited stepwise bubble growth, AW‐PTLs continuously released bubbles due to facile gas permeation. The pressure profiles showed that the pressure on the pristine NF increased to ≈1070 Pa, then dropped to ≈400 Pa as the bubble was ejected (Figure 3e; Figure S15, Supporting Information). This stick‐slip‐like cycle of pressure build‐up and bubble release was repeated, potentially leading to bubble accumulation between the catalyst layers and PTLs, which increases overpotential in water electrolyzers. In contrast, the pressure for AW‐PTLs increased to ≈100 Pa, then dropped and maintained ≈20 Pa, resulting in steady bubble release. The pristine NF had an average BPP—the pressure at which bubbles are ejected through substrates—of 1006.3 Pa (Figure 3f), comparable to its theoretical capillary pressure (974.8 Pa, detailed in the Supporting Information). In contrast, AW‐PTLs exhibited a significantly reduced average BPP of 64.8 Pa in the forward direction. This value was cross‐validated with the bubble‐absorbing pressure (49.5 Pa) (Figure S16, Supporting Information). In the reverse direction, the measured BPP (1031.4 Pa) was similar to that of the pristine NF, as the bubble still needed to overcome the capillary pressure of the bare Ni side. Similar trends of a significant reduction in BPP after the PTFE modification were observed for other PTLs (Figure 3g), demonstrating the general applicability of our approach.

### 2.3 Effect of AW‐PTLs on the Performance of AEMWEs

**Figure 4:**
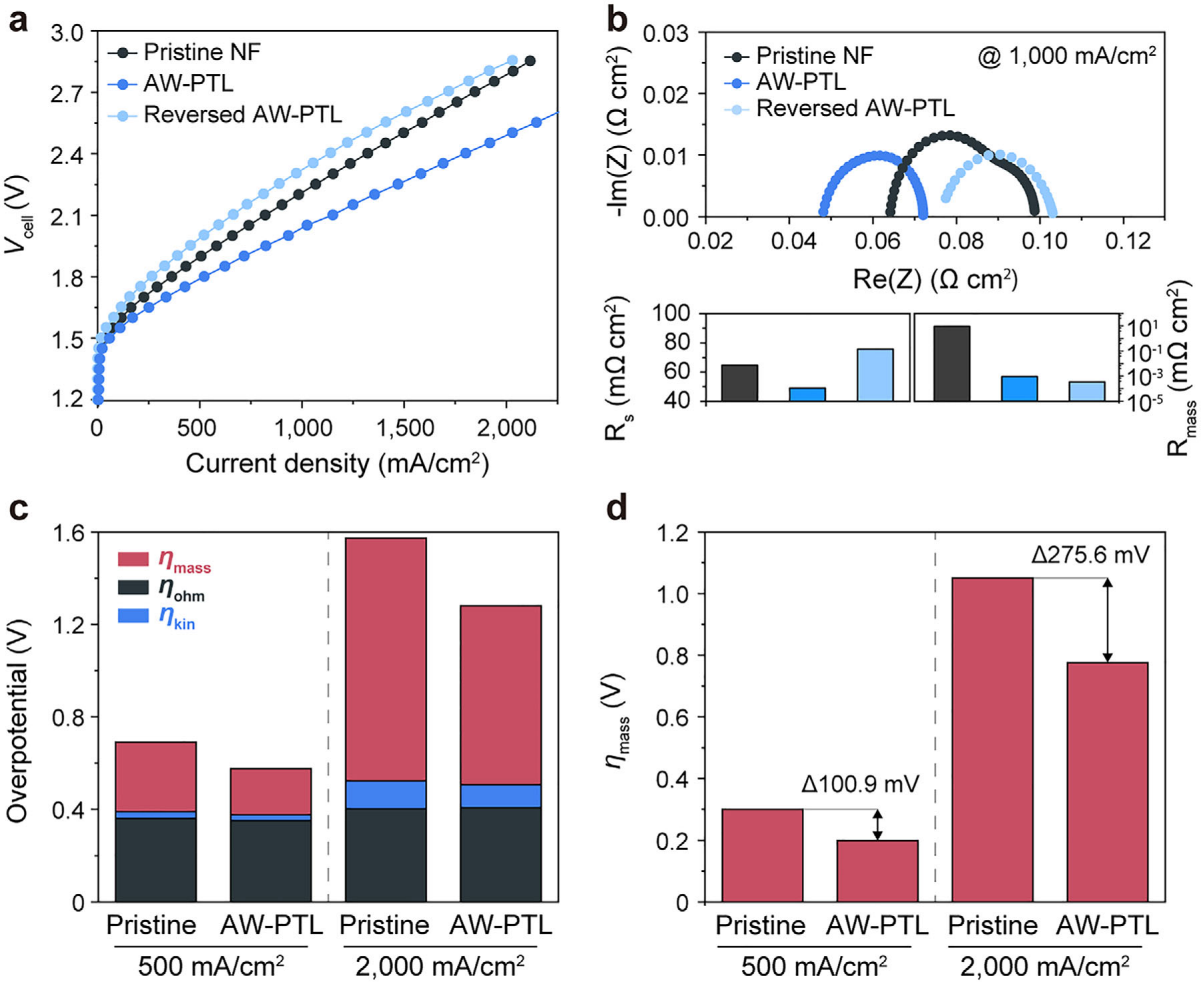
Effect of AW‐PTLs on the performance of AEMWEs. a) LSV curves of AEMWEs with pristine NF, AW‐PTLs, and reversed AW‐PTLs. All electrochemical measurements were conducted in a two‐electrode, zero‐gap AEMWE configuration, and all voltages reported correspond to total cell voltages (*V*_cell_) without iR correction. b) Impedance spectra of AEMWEs using NF, AW‐PTLs, and reversed AW‐PTLs at 1000 mA cm^−2^. The lower panels summarize the extracted solution resistance (*R*_s_) and mass transport resistance (*R*_mass_), highlighting the mitigation of transport limitations by AW‐PTLs. c) Overpotential breakdown analysis of the respective AEMWEs at 500 and 2000 mA cm^−2^. d) Comparison of mass transport overpotentials in the respective AEMWEs.

We investigated the effect of AW‐PTLs on the performance of AEMWEs using 1 m KOH as the electrolyte. For practical electrolyzer applications, AW‐PTLs were prepared by modifying only one side of the upper half of the NFs with PTFE to 1) ensure electrolyte supply to the catalysts and 2) facilitate gas bubble removal. Of note, current densities were calculated with respect to geometric surface area. AEMWEs with AW‐PTLs exhibited significantly improved performance, achieving a current density of 1151 mA cm^−2^ at 2.1 V—a 1.4‐fold increase compared to pristine NFs (**Figure** **4**a; Table S3, Supporting Information). Furthermore, the advantageous bubble‐management effect of AW‐PTLs was retained even under industrially relevant conditions, as demonstrated by stable operation in 3 m KOH at 70 °C (Figure S17, Supporting Information). We also found that AW‐PTLs were effective on both the cathode and anode, with the best performance achieved when used on both sides (Figure S18, Supporting Information). The cell voltage difference between AEMWEs using pristine NFs and those with AW‐PTLs increased with current density, likely due to gas bubble accumulation that led to performance degradation in the absence of AW‐PTLs. Interestingly, the performance of AEMWEs with AW‐PTLs was lower than that of those with pristine NFs when the PTFE‐coated side faced the catalyst layers (i.e., in the reverse direction) (Figure 4a). Moreover, when NFs were fully covered with PTFE on one side, AEMWE performance drastically declined (Figure S19, Supporting Information). Given that capacitance showed a slight decrease as the coverage of hydrophobic materials increased, most of the electrochemically active surface area (ECSA) originates from the nanostructured electrocatalysts rather than the PTL itself (Figure S20, Supporting Information). Because the PTFE coating was applied exclusively to the PTL region, which does not contain nanostructured catalysts, the catalyst layer remained uncoated and fully accessible, resulting in only ≈9% decrease in ECSA despite the surface modification. Combined together, these results can be attributed to 1) gas bubble trapping and ineffective electrolyte supply, and 2) poor electrical contact between the catalyst layers and PTLs.

### 2.4 Overpotential Breakdown Analysis in AEMWEs With and Without AW‐PTLs

To understand the role of AW‐PTLs in performance improvement, we conducted electrochemical impedance spectroscopy (EIS) at a current density of 1000 mA cm^−2^, where vigorous bubble formation occurs. Both ohmic and charge transfer resistances were reduced when using AW‐PTLs compared to conventional NF PTLs (Figure 4b). When tested in reversed orientation (with the PTFE‐coated side facing the catalyst layer), the series resistance (*R*_s_) increased slightly, indicating additional interfacial resistance. Nevertheless, the mass transport resistance (*R*_mass_) remained significantly lower than that of pristine NF, confirming that anisotropic wettability continues to promote efficient bubble removal. These results demonstrate the necessity of dual‐wettability configurations, in which hydrophilic Ni domains ensure ionic and electronic transport, while hydrophobic domains facilitate liquid‐gas diffusion. Consistent with this, pristine NF showed an additional semicircle at low frequencies, indicating poor diffusion and, therefore, inefficient removal of gaseous products. Given that both pristine NF and AW‐PTLs provide identical electrical contact to the catalysts, with all other conditions being equal, and both resistances are influenced by the effective cross‐sectional area of the catalyst‐electrolyte interface, the observed reduction in both resistances can be attributed to the mitigation of gas bubble trapping when using AW‐PTLs, leading to lower resistances and improved efficiency.

Next, we conducted an overpotential breakdown analysis of AEMWEs with and without AW‐PTLs (Figure 4c,d). The total overpotential (*η*_total_) in electrochemical systems consists of ohmic (*η*_ohm_), kinetic (*η*_kin_), and mass transport (*η*_mass_) overpotentials.^[^ ^46^, ^47^ ^]^ Specifically, *η*_ohm_ was obtained from the high‐frequency resistance measured by EIS, *η*_kin_ was determined from Tafel extrapolation at low current densities, and *η*_mass_ was calculated as the residual between *η*_total_ and the sum of *η*_ohm_ and *η*_kin_. While *η*_kin_ is closely related to the intrinsic properties of electrocatalysts and dominates at low current densities, *η*_ohm_ and *η*_mass_ become increasingly important at higher current densities due to ionic resistance and bubble‐induced transport limitations. Although *η*_kin_ accounted for a large portion of the total overpotential across all current ranges, its increase was less significant compared with *η*_mass_ (Figure 4c). For instance, when the current density increased from 500 to 2000 mA cm^−2^, the pristine NF exhibited variations in kinetic (Δ*η*_kin_) and ohmic (Δ*η*_ohm_) overpotentials of 41.68 and 90.84 mV, respectively, while the corresponding variation in mass transport overpotential (Δ*η*_mass_) was 750.33 mV. The dominance of *η*_mass_ at higher current densities highlights the importance of bubble manipulation under practical hydrogen production conditions. As expected, AW‐PTLs led to a significant reduction in overpotentials compared to pristine NF with a prominent reduction in *η*_mass_ than in *η*_ohm_. Specifically, AW‐PTLs reduced Δ*η*_mass_ by ≈100.9 and 275.6 mV compared to pristine NF at current densities of 500 and 2000 mA cm^−2^, respectively. Furthermore, AW‐PTLs suppressed potential fluctuations during stepwise constant voltage operations (Figure S21, Supporting Information). Collectively, these results suggest that the performance improvement achieved with AW‐PTLs is primarily due to effective bubble removal and enhanced mass transport.

### 2.5 In Situ Observation of Bubble Dynamics in AEMWEs

**Figure 5:**
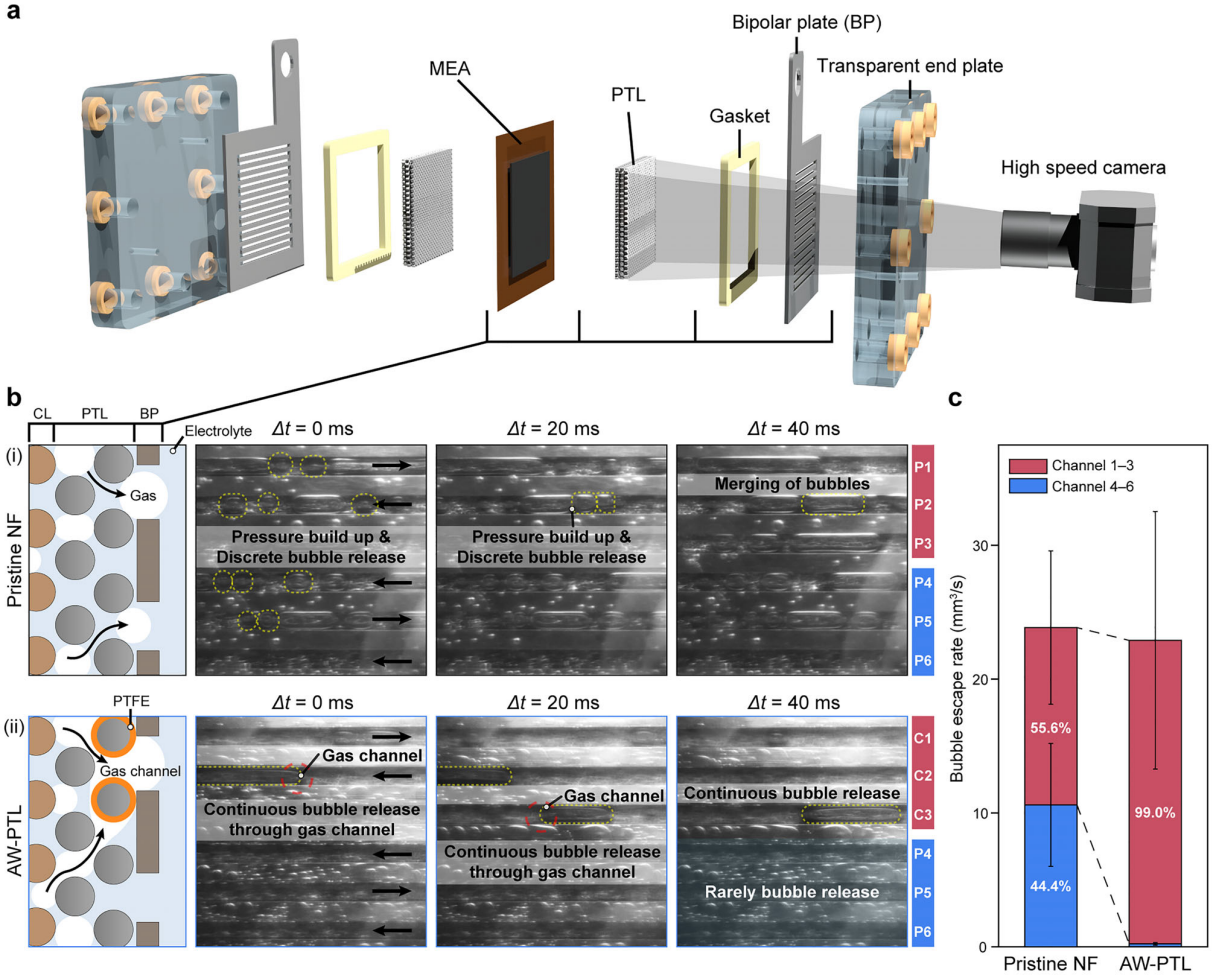
In situ observation of bubble flows in AEMWEs using pristine NF and AW‐PTLs. a) Experimental setup with a custom‐made transparent cell. b) (left) Schematic illustrations showing the bubble release mechanisms in the respective AEMWEs. (right) Snapshots of the anode‐side bubble flow channels at 2000 mA cm^−^^2^ for AEMWEs using i) pristine NF and ii) AW‐PTLs. Yellow dashed circles mark bubbles that were actually observed to be ejected during the video recordings. Spherical bubbles were discretely released through pristine NF, whereas cylindrical bubbles were continuously released through AW‐PTLs. c) Comparison of bubble escape rate for the respective AEMWEs.

To directly observe enhanced bubble flows in AEMWEs using AW‐PTLs, we designed a custom‐made transparent AEMWE cell (**Figure** **5**a). Six serpentine channels located in the center of the cell were monitored during water electrolysis: the top three near the outlet and the bottom three near the inlet. The channels were numbered sequentially from the top, with “P” representing the pristine part and “C” representing the PTFE‐coated part. For pristine NFs, individual bubbles were sparsely ejected in spherical shapes and merged into short cylindrical bubbles (Figure 5b; Movies S5 and S6, Supporting Information). This behavior was consistently observed across all channels (P1–P6). In contrast, for AW‐PTLs, long cylindrical bubbles were continuously ejected. Interestingly, bubble ejections were predominantly observed in the channels near the PTFE‐coated region (C1–C3), with only a few spherical bubbles present in the unmodified region (P4–P6). In summary, the pristine NF exhibited a repeated cycle of single‐bubble ejection and merging across all channels, whereas the AW‐PTL showed continuous bubble ejection primarily in channels near the PTFE‐coated region. For quantitative comparison, the bubble escape rate was calculated, as detailed in Note S3 (Supporting Information). At a constant current density of 2000 mA cm^−2^, the overall bubble escape rates were nearly identical for both pristine NF and AW‐PTL. However, the bubble escape rates varied significantly depending on the channel positions and the type of PTL (Figure S22, Supporting Information). For pristine NF, a slightly larger amount of the bubbles was released through the upper P1–P3 channels (55.6%) due to buoyancy. In contrast, 99% of bubbles escaped near the PTFE‐coated C1–C3 channels for AW‐PTLs. These differences can be attributed to the significant reduction in BPP for AW‐PTLs compared to pristine NF. For pristine NF, bubbles were often trapped between the catalyst layer and the NF PTL, leading to discontinuous ejection through the pristine channels. In AW‐PTLs, however, bubbles near the P4–P6 region were compressed but efficiently spread to the PTFE‐coated C1–C3 region rather than being released through the unmodified NF. As a result, most of the bubbles were smoothly released through the PTFE‐coated region. These results further support our conclusion that the observed performance improvement is due to the enhanced gas bubble removal by AW‐PTLs.

### 2.6 Applicability of AW‐PTLs in AEMWEs

**Figure 6:**
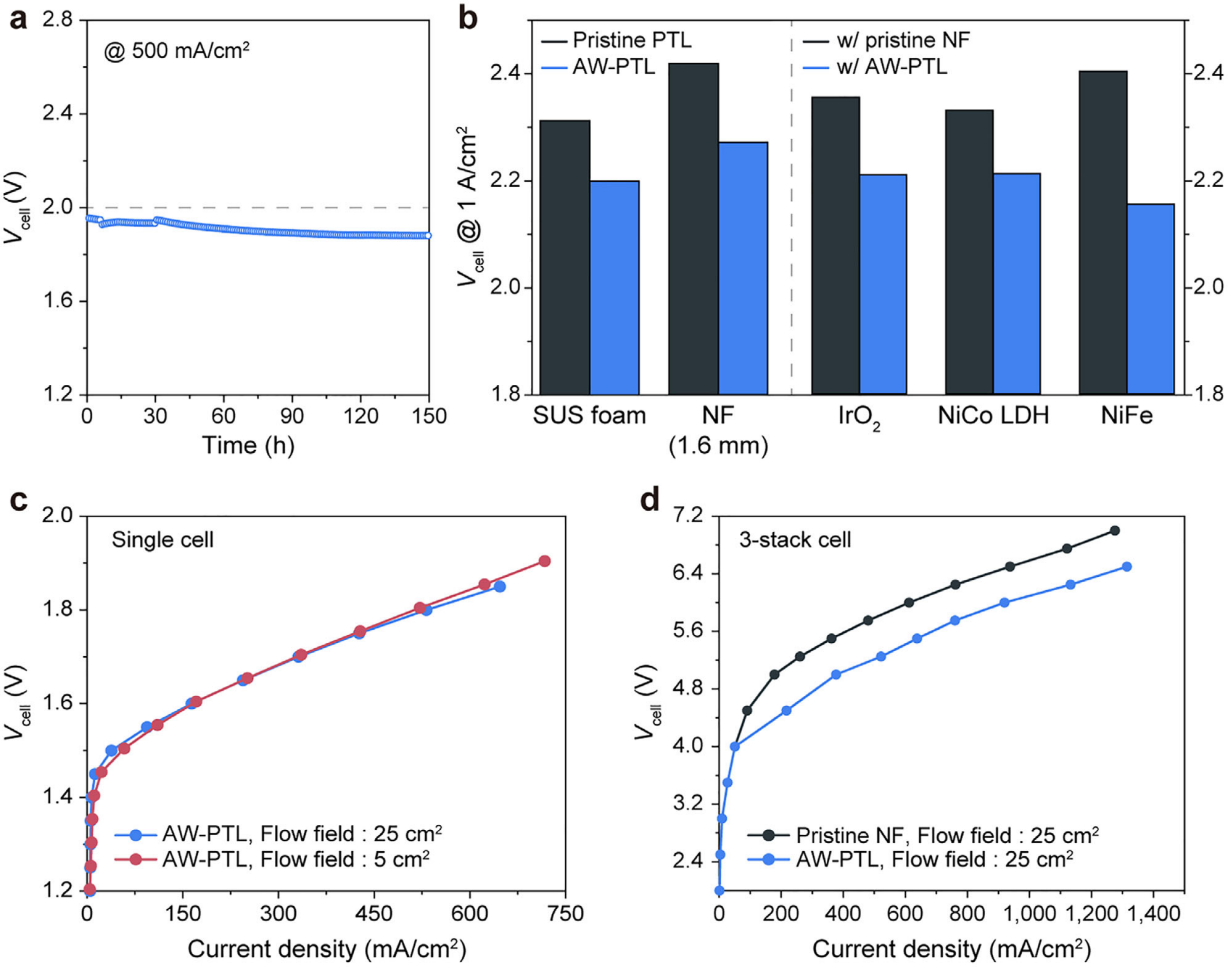
Applicability of AW‐PTLs. a) Stability tests at 500 mA cm^−2^ for 150 h. b) Versatility testing of AW‐PTLs. (left) Application of the AW‐PTLs to 1.6 mm thick NF and SUS foam. (right) Comparison of cell voltage at 2000 mA cm^−2^ for various electrocatalysts, including IrO_2_, NiCo LDH, and NiFe, using pristine NF and AW‐PTL. c) Polarization curves of the large‐sized single cell with a flow field of 25 and 5 cm^2^. d) LSV curves for overall water splitting with pristine and AW‐PTL at 55 °C with 3‐stack electrolyzers.

We conducted long‐term chronopotentiometry (CP) to demonstrate the stability of AW‐PTLs (**Figure** **6**a). Prolonged CP analysis at a current density of 500 mA cm^−2^ at 55 °C showed that the cell voltage remained stable for at least 150 h, and even under 1000 mA cm^−2^ the stability was retained for 250 h (Figure S23, Supporting Information). The slight decrease in potential observed during initial hours is likely due to ionomer channel conditioning and stabilization of the electrolyte‐ionomer interface.^[^ ^48^ ^]^ After prolonged operation, static contact angle measurements indicated that the surface retained its superhydrophobicity even after 150 h of testing (Figure S24, Supporting Information). Additionally, electron microscopy confirmed that the PTFE in AW‐PTLs maintained its mechanical interlocking with the Ni framework (Figure S25, Supporting Information). In parallel, the peel strength—a unique material property reflecting interfacial adhesion between an adhesive and the material of interest, rather than bulk mechanical strength—of the AW‐PTL was significantly lower than that of pristine NF due to the intrinsically low surface energy of the PTFE layer. Notably, the peel strength of AW‐PTL after operation remained nearly unchanged, even after five attach‐peel cycles (Figure S26, Supporting Information), validating the mechanical robustness of the PTFE layer. These findings demonstrate that AW‐PTLs can withstand harsh alkaline conditions and continuous bubble‐induced mechanical stress. In principle, intermediate radical species could induce electrochemical scission of polymeric materials and affect the long‐term stability of AW‐PTLs; however, other functional components (e.g., ion‐selective membranes and ionomers) are expected to degrade more rapidly, underscoring the relative robustness of PTFE and AW‐PTLs.

We further validated our approach by applying it to various PTL materials and catalysts. Thicker NF (1.6 mm thick) and SUS foam were used to fabricate AW‐PTLs, and efficiency improvements were observed in all cases (Figure 6b; Figure S27, Supporting Information). Furthermore, AW‐PTLs were tested with benchmark OER catalysts, such as NiFe, IrO_2_, and NiCo layered double hydroxide (NiCo LDH), and the approach proved effective regardless of the catalyst's type (Figure 6b; Figures S28 and S29, Supporting Information).

Lastly, we tested the scalability of our approach by fabricating large‐area and short‐stack AEMWEs (Figure 6c,d). First, we confirmed that a single cell with large area AW‐PTLs up to 25 cm^2^ maintained its activity, demonstrating the continued benefit of bubble manipulation even for large‐area electrolyzers (Figure S30, Supporting Information). For example, 5 and 25 cm^2^ AEMWE single cells showed current densities of 521.73 and 531.78 mA cm^−2^ at 1.8 V. Additionally, in a 3‐stack cell configuration, AW‐PTLs exhibited enhanced performance, achieving a current density of 919.65 mA cm^−2^ at 6 V—significantly higher than the 612.15 mA cm^−2^ achieved with pristine NF. These results consistently demonstrate the stability, general applicability, and scalability of our approach, highlighting its potential for practical application.

## 3 Discussions

Our development of AW‐PTLs marks a significant advancement in the practical application of water electrolyzers. Unlike traditional PTLs that are fully hydrophilic, AW‐PTLs strategically combine hydrophilic and hydrophobic regions to enable directional transport of liquid electrolytes and gaseous products. This innovation addresses the critical challenge of gas bubble accumulation in dynamic three‐phase conditions, which has limited efficiency and long‐term operational stability in electrolyzers. While we have demonstrated their effectiveness only in AEMWEs, AW‐PTLs can, in principle, be effectively utilized in other types of water electrolyzers, including conventional alkaline water electrolyzers and next generation systems such as PEMWEs and BPMWEs.

First, the effectiveness of AW‐PTLs should be validated across electrochemical platforms. Diverse gas‐involving systems, such as BPMWEs, CO_2_ electrolyzers,^[^ ^49^, ^50^, ^51^ ^]^ and hydrogen fuel cells (PEMFCs), would confirm the broad applicability of this approach. Extending AW‐PTLs to these systems could significantly enhance performance by improving reactant mass transport and fluidic management, potentially revolutionizing these technologies. Next, exploring alternative hydrophobic coatings or deposition materials that avoid perfluoroalkyl and polyfluoroalkyl substances (PFAS) might be necessary. Developing eco‐friendly materials or methods would improve the environmental sustainability of this approach,^[^ ^52^, ^53^, ^54^ ^]^ aligning it with global efforts to reduce reliance on environmentally persistent chemicals. Finally, fundamental research on bubble dynamics within the PTL is crucial for optimizing the hydrophobic and hydrophilic regions and their geometry. Because gas‐involving electrochemical platforms inherently operate under transient three‐phase microenvironments, it is particularly important to understand how bubble transport rates across dual‐wetting substrates influence ionic transport.^[^ ^55^, ^56^, ^57^, ^58^, ^59^, ^60^, ^61^ ^]^ Clarifying these correlations between dynamic wetting kinetics and electrochemical behavior will provide a stronger mechanistic foundation for the rational design of next‐generation PTLs.

## 4 Conclusion

In this work, we introduced a novel strategy to enhance the efficiency of AEMWEs using AW‐PTLs. The AW‐PTLs were readily prepared by partially coating one side of hydrophilic Ni foam with hydrophobic PTFE, enabling unidirectional transport of liquid electrolytes and gaseous products. This design effectively facilitated gas bubble removal, significantly reducing the BPP of the Ni foam from 1006.3 to 64.8 Pa. Electrochemical tests demonstrated that AEMWEs using AW‐PTLs exhibited enhanced performance, including a reduction in mass transport overpotential by 275.6 mV at 2000 mA cm^−2^ and stable operation for over 150 h at 500 mA cm^−2^. Additionally, in situ bubble dynamics observations validated the continuous bubble removal mechanism. Unlike conventional studies that primarily focus on the design and synthesis of novel electrocatalysts and membranes, our work shifts the paradigm by highlighting the crucial role of multi‐wettable PTL design in electrolyzer performance through the regulation of extrinsic activity.

## Conflict of Interest

Y.K., S.L., D.W.L., and J.R. are inventors on a pending PCT patent application (no. PCT/KR2024/000469), filed on January 10, 2024, related to the PTL designs for water electrolysis presented in this work.

## Author Contributions

Y.K. and S.L. contributed equally to this work. Y.K., S.L., D.W.L., and J.R. designed and conceptualized the fundamental idea and experiments. D.W.L. and J. R. supervised the overall project. Y.K., J.L., and S.L. contributed to sample preparation, analyze data, conduct experiments, and write the first draft of the paper. Y.K., S.L., J.L., H.K., and G.H.C. designed water electrolysis cells and performed electrochemical reactions. S.L. and G.L. measured and interpreted wetting properties and in situ bubble dynamics. All authors read and commented on the manuscripts.

## Supporting information



Supporting Information

Supplemental Movie 1

Supplemental Movie 2

Supplemental Movie 3

Supplemental Movie 4

Supplemental Movie 5

Supplemental Movie 6

## Data Availability

The data that support the findings of this study are available from the corresponding author upon reasonable request.
