# Supplementary material for: Anisotropically Wettable Porous Transport Layers for Gas Management in Water Electrolyzers
Source: Adv Sci (Weinh). 2025 Nov 8;13(8):e08569. doi: 10.1002/advs.202508569 (PMC12884718; doi:10.1002/advs.202508569)
Supplement: Supplementary file 1 — Supporting Information [file ADVS-13-e08569-s006.pdf]

Supporting Information

Anisotropically Wettable Porous Transport Layers for Gas Management in Water Electrolyzers

Yunseok Kang, Seunghyun Lee, Jinseo Lee, Soi Lee, Geonwoo Lee, Hyeongoo Kim, Gwan Hyun Choi, Jungki Ryu, and Dong Woog Lee**

Y. Kang, S. Lee, J. Lee, S. Lee, G. Lee, H. Kim, J. Ryu, D. W. Lee
School of Energy and Chemical Engineering
Ulsan National Institute of Science and Technology (UNIST)
Ulsan 44919, Republic of Korea.
Email: jryu@unist.ac.kr; dongwoog.lee@unist.ac.kr

Y. Kang, J. Lee, S. Lee, G. Lee, H. Kim, J. Ryu
Emergent Hydrogen Technology R&D Center
Ulsan National Institute of Science and Technology (UNIST)
Ulsan 44919, Republic of Korea.

J. Ryu
Graduate School of Carbon Neutrality
Ulsan National Institute of Science and Technology (UNIST)
Ulsan 44919, Republic of Korea.

J. Ryu
Center for Renewable Carbon
Ulsan National Institute of Science and Technology (UNIST)
Ulsan 44919, Republic of Korea.

G. H. Choi
Department of Chemistry
University of California, Berkeley
California 94720, United States

Materials and Methods

Materials

Ni foam (thickness: 0.5 and 1.6 mm) and stainless steel (SUS) foam (thickness: 1 mm) were obtained from MTI Korea. Ni fiber felt (1.6 mm thick) was purchased from AliExpress. Carbon cloth was obtained from Fuel Cell Earth. Potassium hydroxide (85%), hydrochloric acid (≥37%), potassium iodide (99%), cobalt(II) nitrate hexahydrate (≥98%), iridium(IV) oxide(≥99.9%), nickel(II) nitrate hexahydrate(99.999%), iron(III) nitrate nonahydrate (98%), and nickel(II) sulfate hexahydrate (98%) were purchased from Sigma Aldrich (USA). 20 wt% Pt/Vulcan XC-72 carbon, 5 wt% PiperION dispersion, and anion exchange membrane (AEM, X37-50 Grade RT, Dioxide Materials, USA) were purchased from the Fuel Cell Store (USA).

Characterizations

Morphology observation and elemental mapping analysis were conducted using an SU-7000 field emission scanning electron microscope (Hitachi, Japan). Fourier-transform infrared spectroscopy (FT-IR) was performed in attenuated total reflectance (ATR) mode using a Varian 670-IR spectroscopy (Varian Instruments, USA) in the range of 4500–500 cm^{-1}. In situ visualization of bubble dynamics was recorded at 500 fps using a high-speed camera (FASTCAM Mini UX50, Photron, Japan) equipped with a 50x objective lens (Tu Plan fluor EPI, Nikon, Japan). To directly visualize the internal water electrolysis cell, optically transparent end plates and the channel of the current distributor were customized to clearly show bubble movements at the anode PTLs. During visualization, the transparent electrolysis cell was operated at a current density of 2000 mA/cm^2. 3D X-ray computed tomography (3D Micro-CT) was performed underwater using a Micro-CT scanner (Nikon XT H 225, Nikon Metrology, UK). A 5 wt% KI solution was used as the contrast agent for analyzing the internal

phase of porous PTLs.

Fabrication of porous transport layers (PTLs) with anisotropic wettability (AW-PTLs)

PTLs, including Ni foam (NF), SUS foam, and Ni fiber felt, were pretreated with ethanol for 5 min, followed by a wash in 3 M HCl solution and deionized (DI) water for 20 min in an ultrasonic bath to remove oxide layers. The substrates were then dried with N_2 gas. The PTFE coating was carried out in two steps: spray coating and annealing. Except for SUS foam (to avoid pores blockage by PTFE particles), substrates were partially masked with Kapton tape to prevent PTFE deposition and heated to 250 °C (for SUS foam, to 100 °C) on a heating plate for 5 min. A 60 wt% of PTFE dispersion (10 wt% for SUS foam) was sprayed onto the substrates using an airbrush (0.3 mm nozzle, 1 bar) from a constant height of 15 cm to ensure uniformity. This process was repeated three times at 5-min intervals. Subsequently, the PTFE-deposited electrodes were then annealed in a furnace at 370 °C for 10 min (ramp rate of 2 °C/min) and allowed to cool naturally. The resulting AW-PTLs were repeatedly rinsed with ethanol and DI water and dried with N_2 gas.

Preparation of HER and OER electrocatalysts

24 mg of Pt/C was dispersed in a mixture of 0.5 mL of DI water, 1.5 mL of ethanol, and 50 μL of 5 wt% PiperION ionomer dispersion. This mixture was ultrasonicated for at least 6 h at 30 °C to form a homogeneous catalyst ink. The cathode was prepared by applying the Pt/C ink onto carbon cloth, achieving a loading of approximately 2.0 ± 0.1 mg/cm^2. Similarly, 24 mg of IrO_2 was dispersed in a mixture of 1.5 mL of DI water, 0.5 mL of ethanol, and 50 μL of PiperION dispersion. The IrO_2 ink was prepared following the same procedure as for the Pt/C ink and applied onto NF, resulting in a loading of approximately 3.0 ± 0.1 mg/cm^2. NiFe catalysts were deposited onto NF via electrodeposition. A standard three-electrode electrochemical system

was used, comprising NF, Ag/AgCl (3 M KCl), and a Ni plate as the working, reference, and counter electrodes, respectively. The electrodeposition for NiFe, in a solution containing 3 mM $Ni(SO_4)\cdot 6H_2O$ and 3 mM $Fe(NO_3)_3\cdot 9H_2O$, was performed for 5 min at a constant −1.0 V vs. Ag/AgCl. The NiCo LDH film was electrodeposited at −1.0 V vs. Ag/AgCl in a solution containing 2 mM $Ni(NO_3)_2\cdot 6H_2O$ and 4 mM $Co(NO_3)_2\cdot 9H_2O$ in DI water. The resulting electrodes were rinsed several times with water and ethanol and then dried with N_2.

AEMWE cell assembly

An AEMWE single cell with a flow field area of 5 cm^2 was used for electrochemical analysis. The cell components included a membrane electrode assembly (MEA), a porous transport layer (PTL), and a Ni-based bipolar plate with a single serpentine flow field. Current collectors made of gold-plated Ni and a multi-pass serpentine flow field of 25 cm^2 were employed for both the large-sized single cell and the 3-stack electrolysis cell. Commercial AEMs served as solid electrolytes in the MEA, separating the anode and cathode. The AEMs were conditioned by immersion in 1 M KOH overnight to convert from Cl^- to the OH^- form, followed by rinsing with deionized (DI) water before assembly. After conditioning, the membranes were stored in DI water to prevent drying and CO_2 contamination. Stainless steel, Ni fiber felt, and NF were assembled as PTLs on both sides. PTFE gaskets of varying thicknesses were adjusted based on the thicknesses of the MEA and PTL to seal the cell. The electrolyte reservoirs' temperature was set to 60 °C and the internal temperature of the single cell was maintained at 55 °C±2 °C. The KOH solution was circulated by a peristaltic pump at a flow rate of 2.5 mL/min.

Electrochemical characterizations

Electrochemical analyses were conducted using an SP-300 potentiostat/galvanostat (Bio-logic Science Instruments, France) equipped with a 5 V/10 A current booster. All electrochemical

measurements were recorded without iR-compensation unless otherwise stated. A 1 M KOH was fed into both the cathodic and anodic compartments at a flow rate of 2.5 mL/min using a peristaltic pump. All the experiments were conducted with cell temperature maintained at 55 °C ± 2 °C. Linear sweep voltammetry (LSV) was performed at a scan rate of 10 mV/s, over the potential range from 1.1 V to 2.8 V, unless otherwise stated. Chronopotentiometry for the long-term stability test was performed at a constant current of 500 mA/cm^2. Galvanostatic electrochemical impedance spectroscopy (GEIS) was carried out at each current density, with frequency ranging from 3 kHz to 1 Hz and an AC amplitude of 100 mA. Electrochemically active surface area (ECSA) was estimated from the electrochemical double-layer capacitance (C_{dl}). Cyclic voltammetry (CV) was carried out by sweeping the cell voltage between 0.6 and 1.0 V, where non-faradaic process occurs. Prior to data collection, 10 conditioning scans were performed to stabilize the charging/discharging behavior. Subsequently, CVs were recorded at scan rates of 10, 20, 60, and 100 mV/s. The capacitive current density was plotted as a function of scan rate, and the slope of the linear fit was taken as C_{dl}. The extracted C_{dl} values were then employed for relative comparison of ECSA. I-V curves for a single cell with a flow field of 25 cm^2 were collected via chronopotentiometry with 120 s holds at each voltage step from 1.0 V to 1.85 V at 0.05 V intervals. Polarization curves for a 3-stack electrolyzer with a flow field of 25 cm^2 were obtained by holding the voltage from 2 V to 6.5 V for 2 min at each step.

Overpotential breakdown analysis

The cell voltage obtained from LSV measurements was divided into four components using the following equation:

$$V_{\text{cell}} = V_{\text{rev}} + \eta_{\text{kin}} + \eta_{\text{ohm}} + \eta_{\text{mass}}$$

where V_{cell} is the cell voltage from LSV measurements, V_{rev} is the thermodynamically reversible

potential, η_{kin} is the kinetic overpotential, η_{ohm} is the ohmic overpotential, and η_{mass} is the mass transport overpotential. The thermodynamically reversible potential (V_{rev}) is temperature-dependent and determined by the following equation:

$$V_{rev} = 1.5184 - 1.5421 * 10^{-3}\, T + 9.523 * 10^{-5}\, T\ln T + 9.84 * 10^{-8}\, T^2$$

where T is the cell temperature in Kelvin. η_{ohm} was calculated using:

$$\eta_{ohm} = i \cdot R_{ohm}$$

where R_{ohm} is obtained from high-frequency resistance in EIS measurements. η_{kin} was calculated using:

$$\eta_{kin} = \mathrm{b}\log(i/i_0)$$

where b is the Tafel slope and i_o is the exchange current density obtained from Tafel plot. The Tafel slope and exchange current density were obtained from iR-compensated polarization curves. η_{mass} was determined by subtracting the thermodynamic voltage and kinetic and ohmic overpotentials from the cell voltage:

$$\eta_{mass} = V_{cell} - V_{rev} - \eta_{kin} - \eta_{ohm}$$

Contact angle measurement

The contact angle was characterized using a goniometer (DSA100, KRÜSS, Germany). Water and air contact angles were determined by placing 6 μL water droplet or air bubble on the surface. For the water sliding angle measurement, a 6 μL water droplet-deposited substrate was tilted at a fixed rate of 1°/s. The dynamic contact angle was measured using the Wilhelmy plate method, as the hydrophilic NF absorbed water droplets. The average value was calculated from at least six measurements to ensure reliability.

Bubble force measurement

The bubble-absorbing force measurement was conducted using custom-made equipment, including a microbalance to record force variation throughout the experiments. An air bubble of approximately 0.5 μL was approached and contracted at a constant velocity of 10 μm/s.

Calculation of the pressure profile

The plain tip of the 1 ml syringe was capped by the PTLs, which were sealed with hot-melt adhesive. The syringe was filled with air and immersed in a water bath to a depth of 1 cm to maintain a constant hydrostatic pressure (P_{hyd} = 98.1 Pa, except atmospheric pressure). Subsequently, the air was compressed at a constant rate (0.2 μl/s) during recording (50 fps). The pressure of the syringe inner was calculated using the time (t). The pressure of the syringe (P_{in}) was calculated using the following equation.

$$P_{in}= \frac{P_{initial}V_{initial}\text{-}P_{out}V_{out}}{V_{in}},$$

where the $P_{initial}$, $V_{initial}$, P_{out}, V_{out}, and V_{in} represent the pressure of the initial state (atmospheric pressure (P_{atm}), 1 atm), the volume of the initial state (1 ml), the pressure of the exterior of the syringe ($P_{hyd} + P_{atm}$), the volume of ejected bubble, and the volume of the syringe, respectively. Therefore, the pressure difference (ΔP) between P_{in} and P_{out} was calculated as shown in Figure 3e. In the pressure profile, the bubble point pressure is the pressure difference at which the first bubble appeared. It can be expressed as the following equation.

$$\text{Bubble point pressure} = \frac{1000}{1000\text{-}0.2t_{on}}P_{atm} - P_{out},$$

where t_{on} represents the onset time at which the bubble was ejected.

Peel strength measurement

A 10 mm × 100 mm strip of 3M 9071 double-coated tape was attached to the PTLs (attached area: 10 mm × 10 mm). The tape was pressed twice with a 2.5 kg hand roller at a speed of 10 mm/s to ensure uniform adhesion. It was then peeled off using a universal testing machine (WL2100C, Withlab, South Korea) at a crosshead speed of 100 mm/min. The peel strength was calculated by averaging the force values over the central 25–75% of the peel length, excluding the initial and final transient regions. All measurements were conducted on five independent samples to ensure reproducibility.

Supplementary note 1: Capillary pressure calculation for pristine NF.

When a capillary tube is immersed in a fluid, a phenomenon known as capillary rise or capillary fall occurs, resulting from the balance between gravitational downforce and the interfacial tension between the tube's surface and the fluid. Specifically, capillary rise occurs when the fluid wets the solid surface (dominated by adhesive forces), while capillary fall occurs with non-wetting fluids (where cohesive forces within the fluid are stronger). This results in a capillary force, quantified as capillary pressure (P_c), which represents the pressure difference between the wetting and non-wetting phases. P_c is calculated using the following equation:

$$P_c = \frac{2\gamma\cos\theta}{r}$$

where γ is the interfacial tension of the fluid, θ is the intrinsic contact angle on the surface, and r is the radius of the tube.

In porous media like NF, capillary pressure is the force required to move a fluid through the material's narrow openings. For instance, in a water-filled NF, capillary pressure refers to the force needed to expel the water. The air pressure must exceed the capillary pressure to penetrate the foam. To determine this pressure, we measured the advancing air contact angle on a Ni plate (122.3°) to estimate the intrinsic contact angle of the NF. Using a mercury porosimeter, we determined the mode value of the pore diameter of pristine NF to be 159.6 μm, corresponding to a radius of 79.8 μm. With the interfacial tension of water (γ) at 72.8 mN/m, the capillary pressure of the NF was calculated as −974.8 Pa. This negative value indicates effective air bubble repulsion, which aligns well with the measured BPP (1006.3 Pa) of the NF, thereby validating the BPP measurement.

Supplementary note 2: Laplace pressure-induced unidirectional bubble penetration.

Hydrophilic NF repels air bubbles because of its capillary pressure, which acts to repel bubbles. However, bubbles can penetrate the NF when a gas film is placed on the opposite side, connecting the bubble and the gas film through a channel. This behavior is driven by the difference in Laplace pressure (P_L) between the bubble (high P_L) and the gas film (low P_L). Laplace pressure, the pressure difference across a curved interface, is given by the Young-Laplace equation:

$$P_{\mathrm{L}} = \gamma \left(\frac{1}{R_1} + \frac{1}{R_2}\right),$$

where γ is the interfacial tension of water, and R_1, and R_2 are the bubble's principal radii of curvature. When the bubble adopts a film-like shape, R_1 and R_2 approach infinity, resulting in negligible Laplace pressure in the gas film. Conversely, when a bubble with a truncated spherical shaped is attached to a solid surface, R_1 and R_2 are equal to R, the radius of the bubble. Consequently, the Laplace pressure of the spherical bubble simplifies to:

$$P_{\mathrm{L}} = \frac{2\gamma}{R}$$

In this manner, the Laplace pressure of the air bubble is significantly larger than that of the gas film. This pressure difference is the driving force that causes air bubbles to spontaneously penetrate the PTFE/Ni region through the formed gas channel.

Supplementary note 3: Calculation of bubble escape rate in AEMWE cell.

To elucidate the different behavior of bubbles in flow channels with pristine NF and AW-PTL, we quantified the ejected bubble volume as the bubble escape rate, measured in mm^3/s. This rate is defined as the volume of bubbles released per unit time (s). We methodically counted event frames in each channel, specifically noting instances where bubbles either grew or were newly ejected.

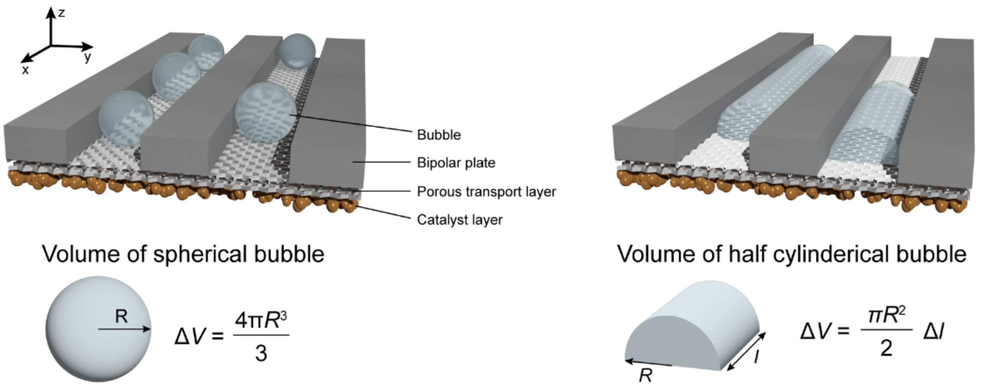

Fig. 1. Illustration of bubble morphologies based on PTL wettability. (left) Spherical bubbles formed on pristine NF and (right) half-cylindrical bubbles formed on AW-PTL.

In the analysis of the pristine NF, we observed that the ejected bubbles were spherical, a morphology attributed to the hydrophilic surface of the pristine NF. By analyzing the radius of these spherical bubbles, measured at 375.2 μm, we calculated an average volume growth rate (ΔV) of 0.2385 mm^3/frame.

Conversely, in the AW-PTL, the ejected bubbles were half-cylindrical. Therefore, we focused on analyzing the length growth rate of the half-cylinder bubbles. Notably, in AW-PTL, bubbles exhibited a preference for spreading in the x-direction rather than in the z-direction, a phenomenon attributed to the presence of a gas film on the PTFE side. The average length growth rate (Δl) for these half-cylindrical bubbles was 617.8 μm, leading to an average volume growth rate of 0.3927 mm^3/frame.

To calculate the average bubble growth rate in mm^3/s, we multiplied the frequency of event frames in each channel (frames/s) by the average volume growth rate (mm^3/frame). This comprehensive approach allowed us to quantitatively compare the bubble behavior in AEMWE cell for the pristine NF and AW-PTL.

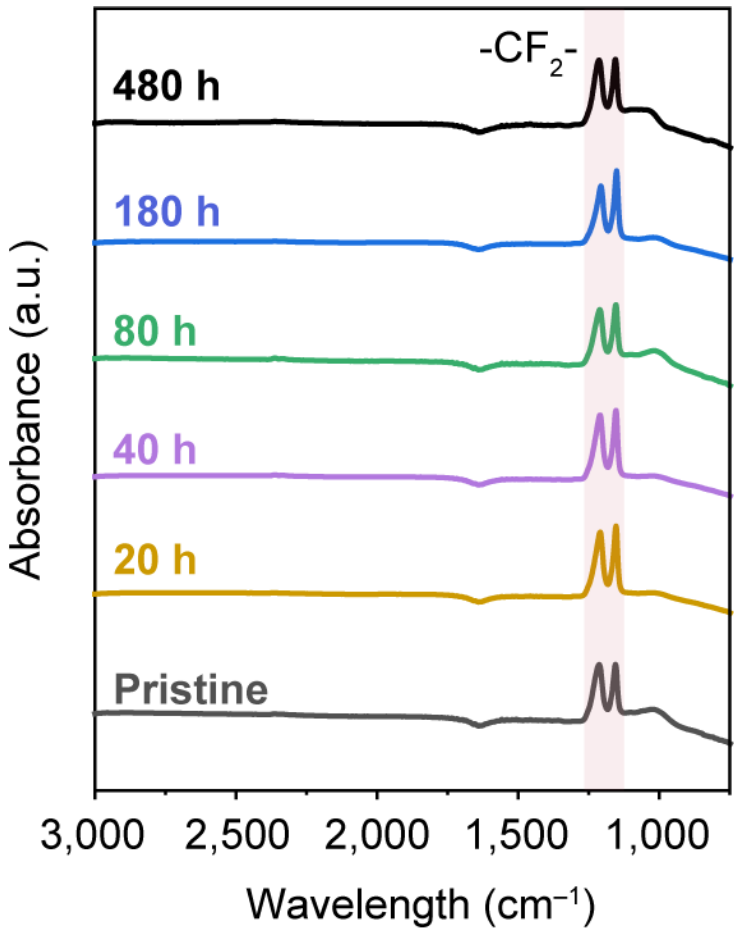

Figure S1. Chemical Stability of PTFE. FT-IR spectra of PTFE were measured after each aging period following immersion in 1 M KOH, with rotation at 900 rpm at 80 °C.

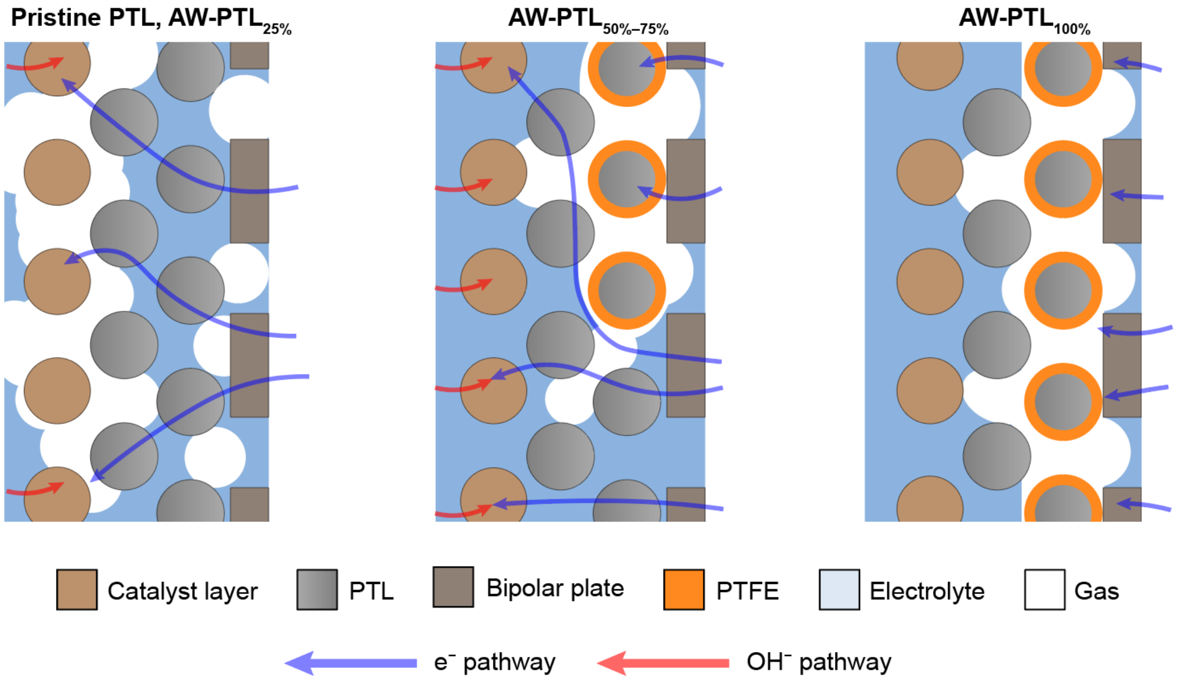

Figure S2. Effect of PTFE coverage ratio on transport properties of PTLs. At low coverage (25%), bubble accumulation hinders ion transport. At moderate coverage (50–75%), dual pathways promote efficient electronic/ionic conduction and bubble release. At full coverage (100%), electrical connectivity is lost because the electron pathway is blocked by the insulating PTFE layers.

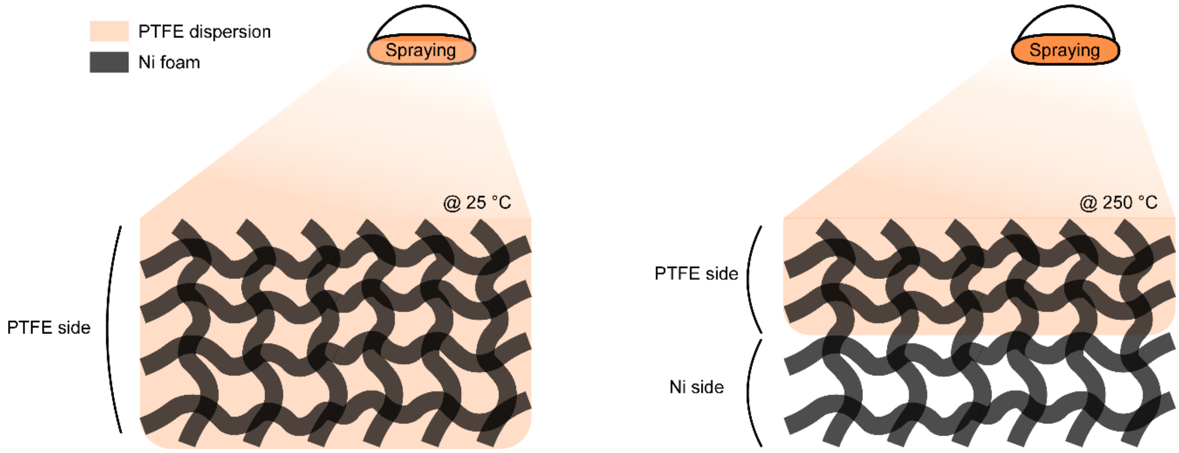

Figure S3. Schematic illustration of the effects of substrate temperature on PTFE deposition in porous media. Variation in the deposition area of NF with substrate temperature during PTFE dispersion spray application. At 250 °C, the dispersion did not fully penetrate, resulting in partial modification, with the rear sections remaining untreated.

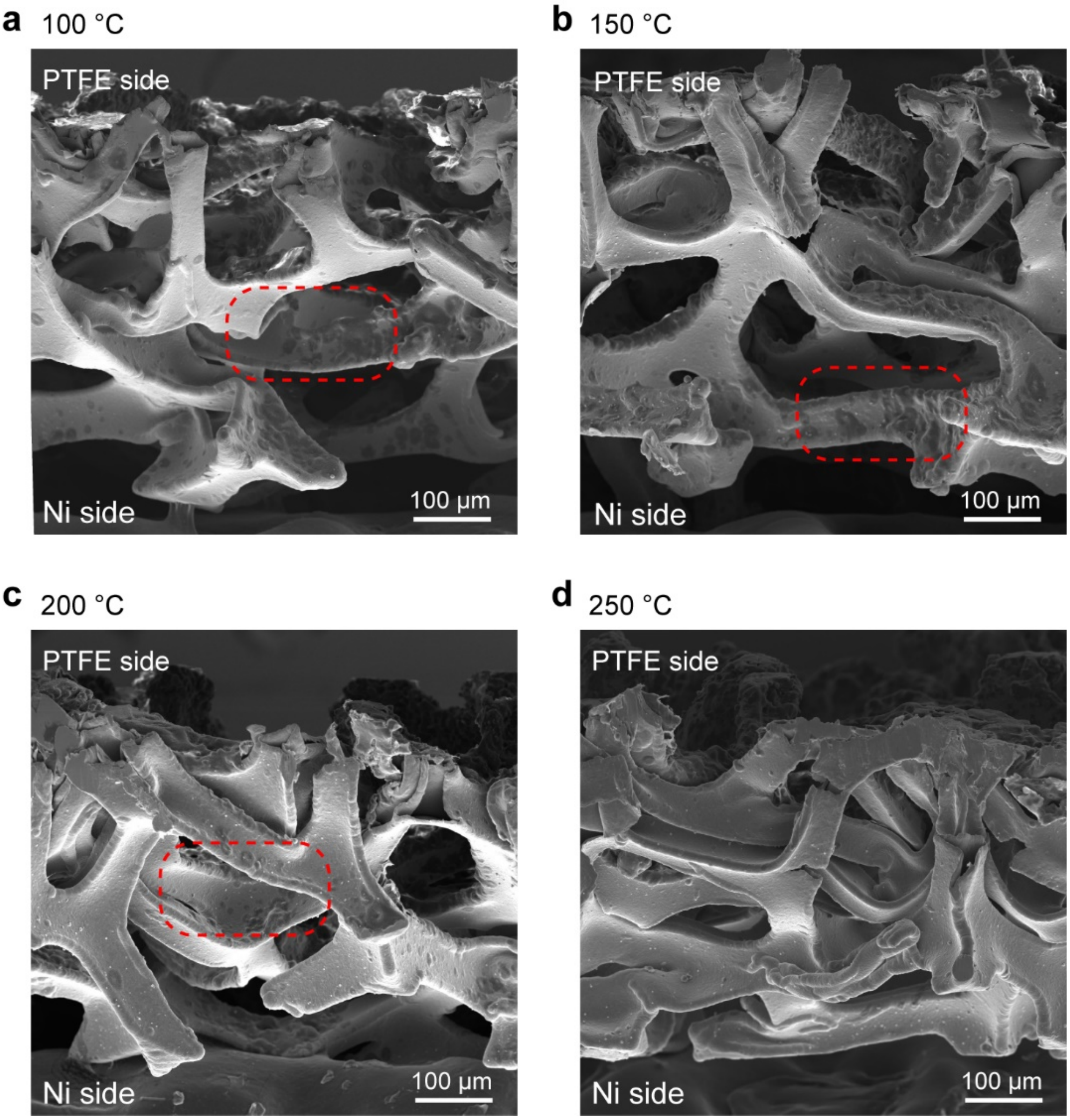

Figure S4. Effects of substrate temperature on PTFE penetration into NF during AW-PTL fabrication. Cross-sectional scanning electron micrographs of AW-PTLs prepared at different substrate temperatures: a) 100 °C, b) 150 °C, c) 200 °C, and d) 250 °C. Red dashed lines indicate the PTFE residues.

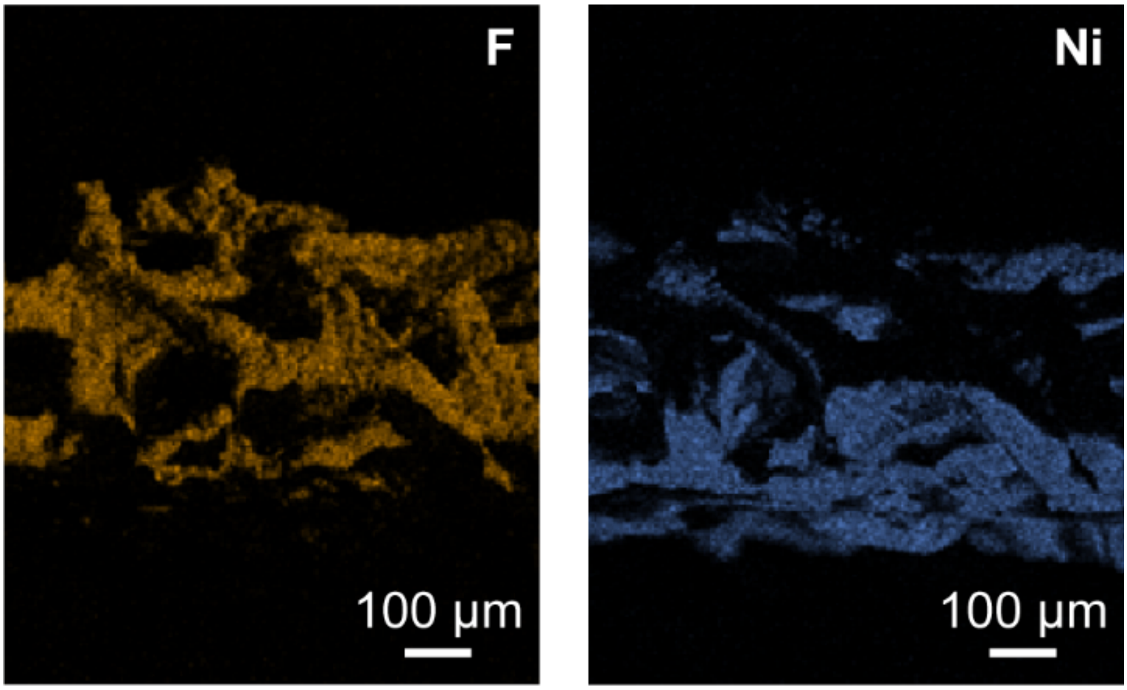

Figure S5. Energy-dispersive X-ray spectroscopy (EDS) analysis of AW-PTL. Cross-sectional EDS mapping images of the AW-PTL showing the distribution F and Ni elements.

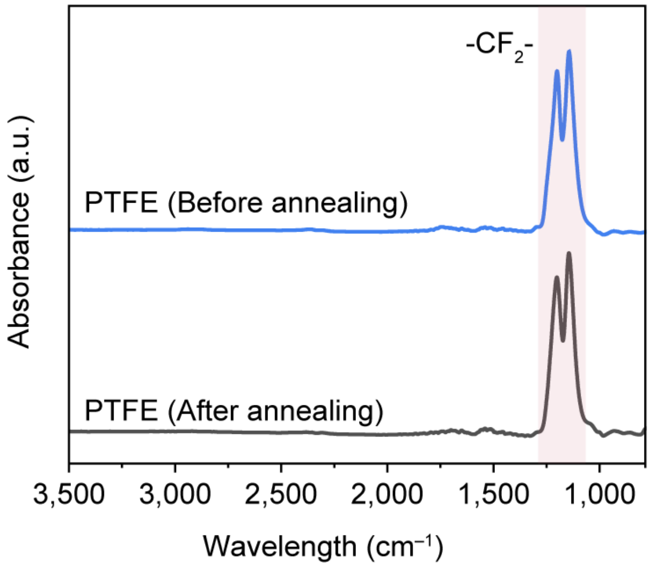

Figure S6. Comparison of FT-IR spectra before and after annealing of PTFE. FT-IR spectra of PTFE before and after annealing during AW-PTL fabrication show that PTFE preserved two characteristic peaks at 1210 and 1153 cm^{-1}, corresponding to the asymmetric and symmetric stretching of the $-CF_2-$ groups, respectively.

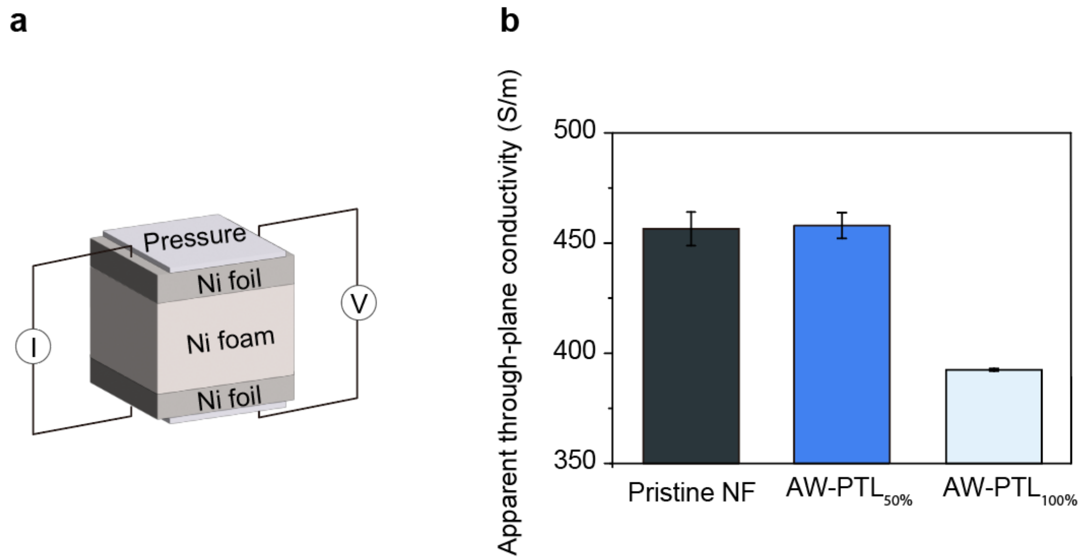

Figure S7. a) Schematic illustration of the through-plane conductivity measurement. b) Conductivity values of pristine NF, AW-PTL$_{50\%}$, AW-PTL$_{100\%}$ confirming that partial PTFE coverage preserves cross-sectional conductivity, whereas complete coverage (100%) significantly hinders electron transport.

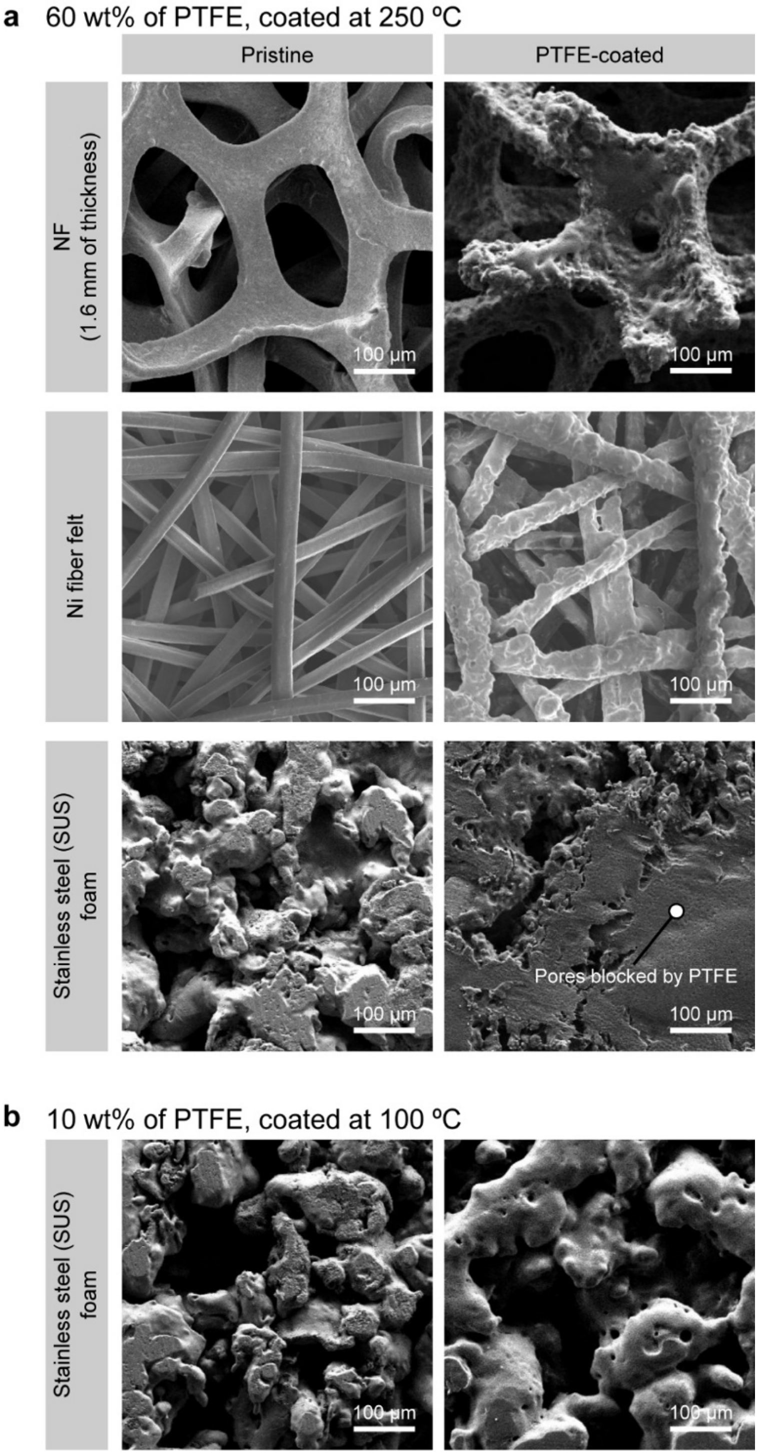

Figure S8. SEM images of various PTLs modified with PTFE under different conditions. a) Top-view SEM images of substrates, including NF (1.6 mm thickness), Ni fiber felt, and SUS foam, all coated with 60 wt% PTFE dispersion at 250 ℃. b) Top-view SEM images of SUS foam coated with 10 wt% PTFE dispersion at 100 ℃. The concentration of PTFE dispersion was diluted to prevent pore blocking by the PTFE particles, and the temperature was reduced to facilitate penetration into the dense porous structure of SUS foam.

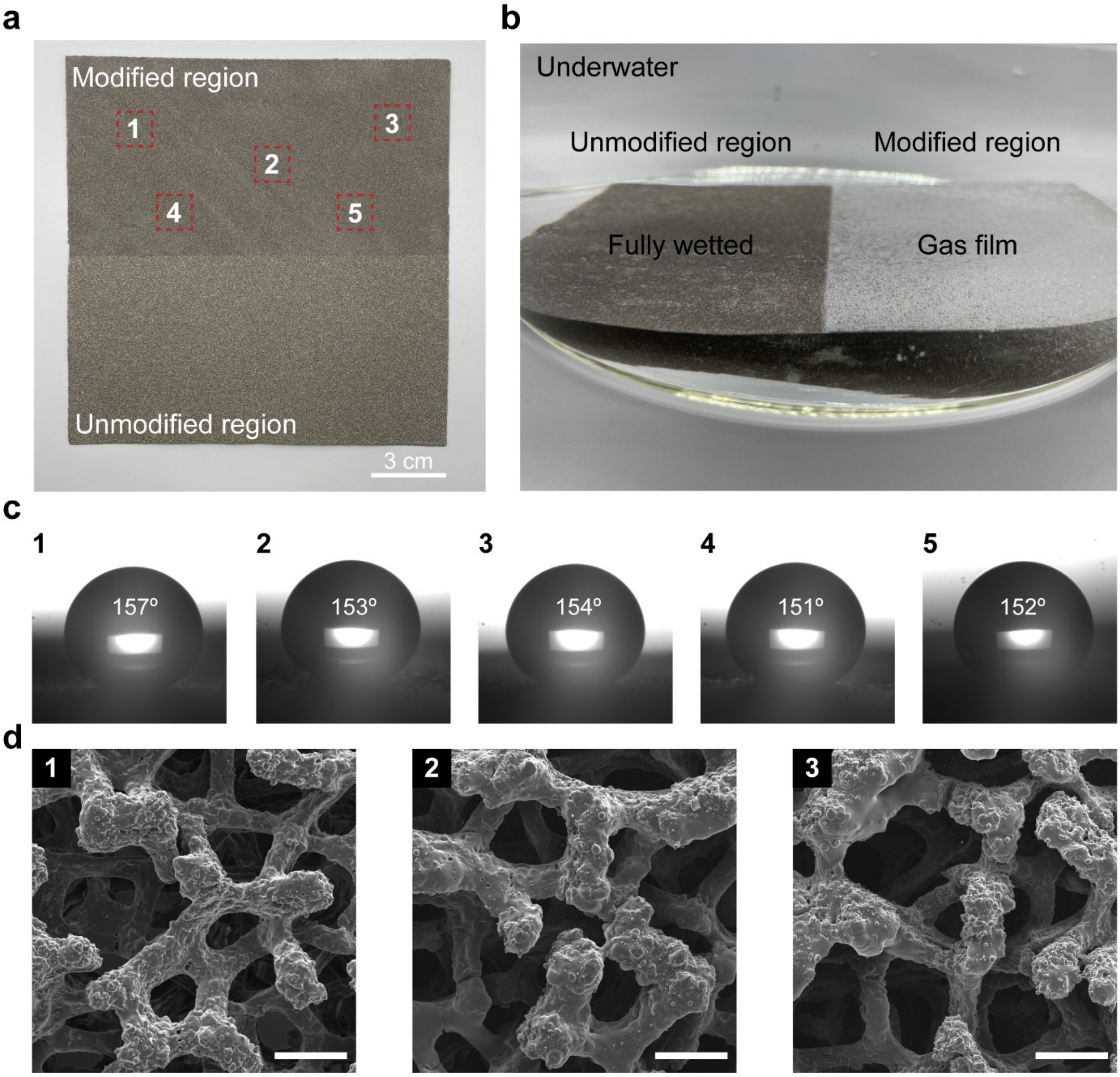

Figure S9. Fabrication of large-area AW-PTL. a) Digital image of a large-size AW-PTL (225 cm^2). b) Image depicting gas layer formation in the PTFE-deposited sections and liquid penetration in the Ni region underwater. c) Measurement of water contact angles. d) Electron micrographs of various positions on the AW-PTL, as marked in a).

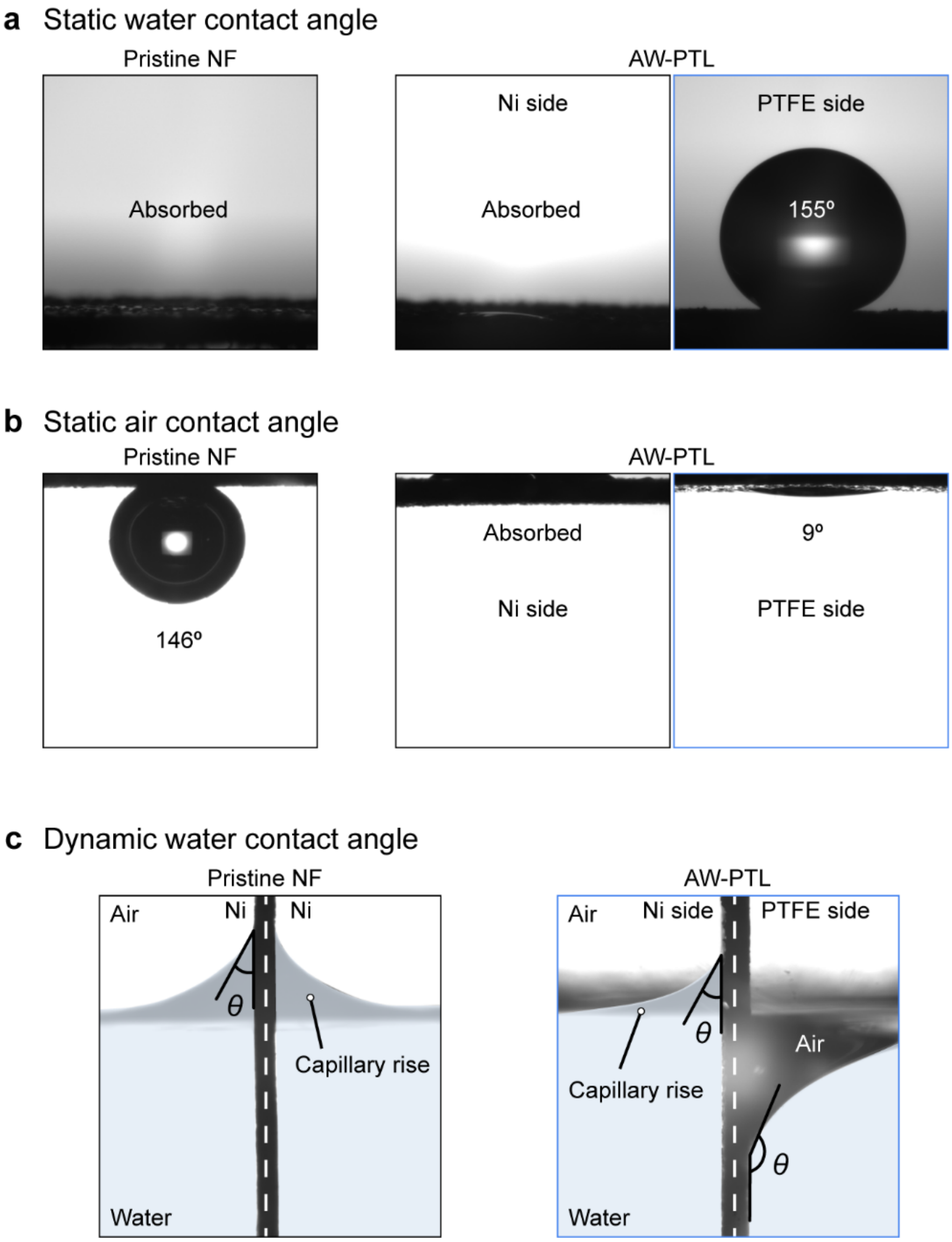

Figure S10. Wettability of pristine NF and AW-PTL. a) Static water contact angle. b) Static air contact angle. c) Dynamic water contact angle.

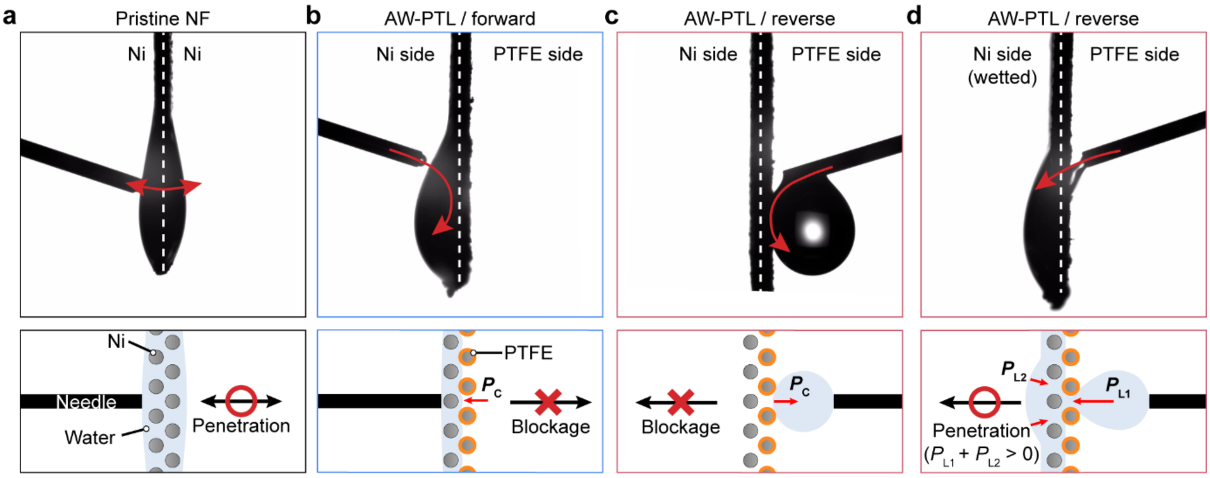

Figure S11. Water permeability test of various PTLs. a) Pristine NF. b) AW-PTL in the forward direction (Ni to PTFE-coated side), c, d) AW-PTL in the reverse direction: c) without and d) with a wetted Ni side. The top row displays photos illustrating water permeability, while the bottom row features schematics depicting the forces acting on a water droplet. In these schematics, P_C, P_{L1}, and P_{L2} denote capillary pressure, Laplace pressure of the water droplet, and Laplace pressure of the water film, respectively.

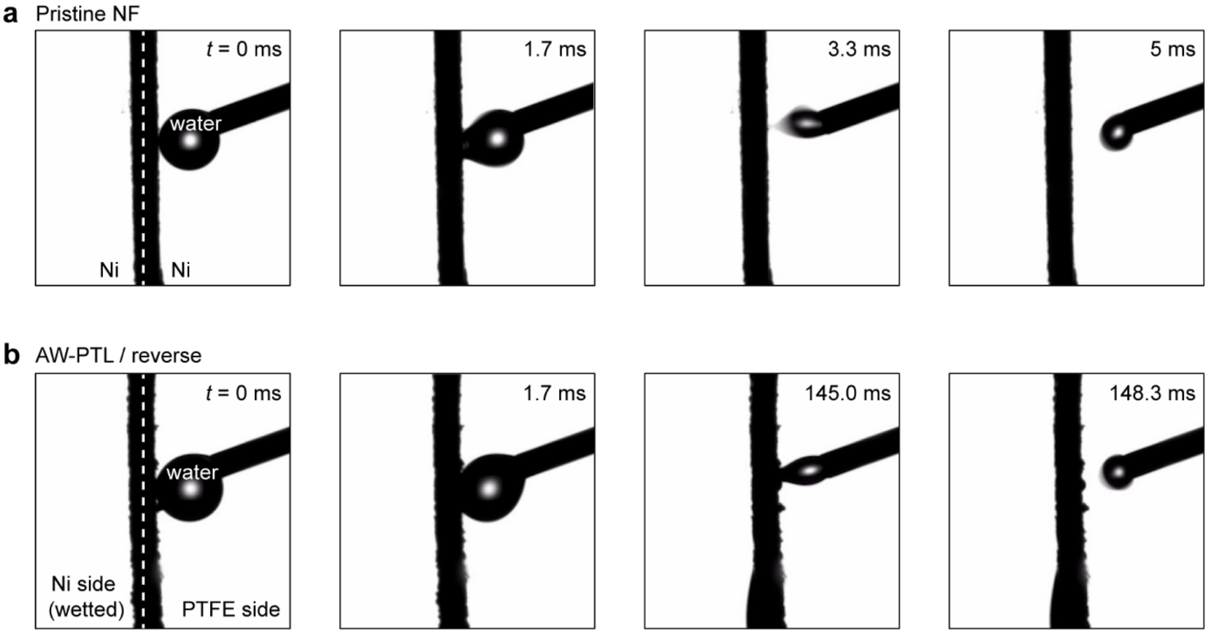

Figure S12. Water absorption time of PTLs. a, b) Photos showing water absorption for a) pristine NF and b) AW-PTL with the wetted Ni side in the reverse direction.

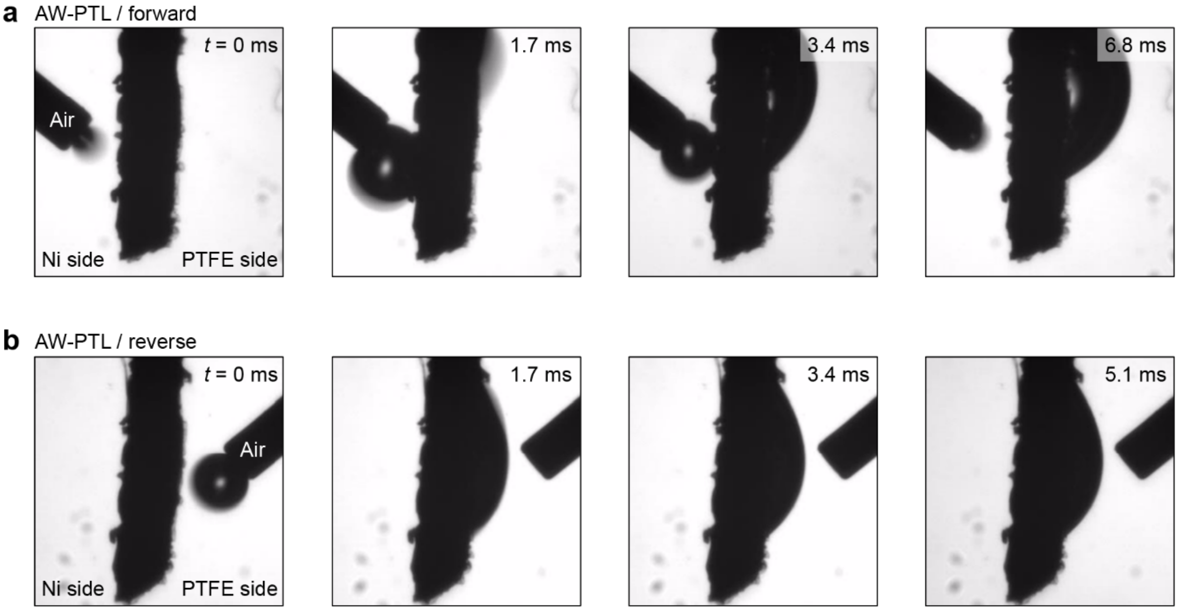

Figure S13. Time-resolved visualization of gas penetration through the AW-PTL in a) the forward direction (Ni side to PTFE side) and b) reverse direction (PTFE side to Ni side).

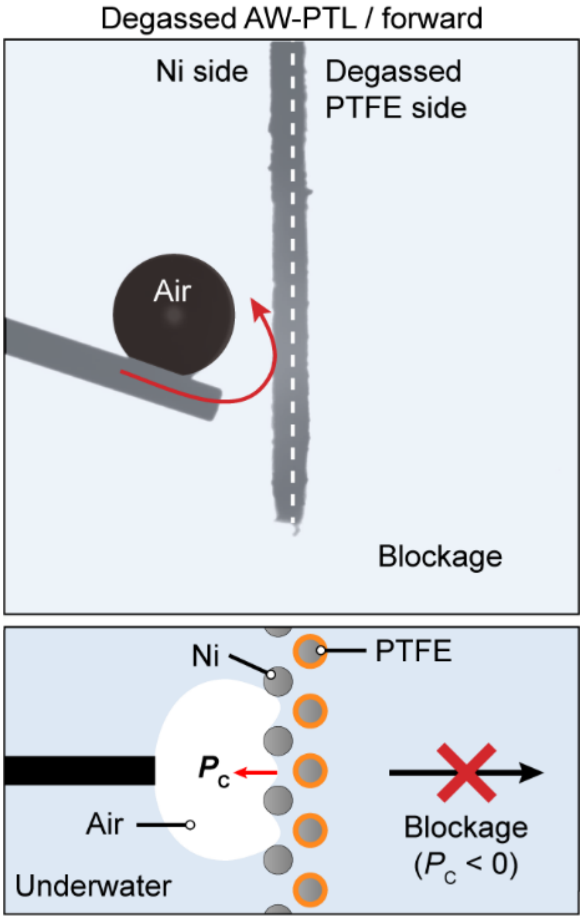

Figure S14. Gas permeability test for degassed AW-PTL. The top row depicts the gas permeability test setup, and the bottom row presents schematics illustrating the forces acting on air bubbles. P_C indicates capillary pressure.

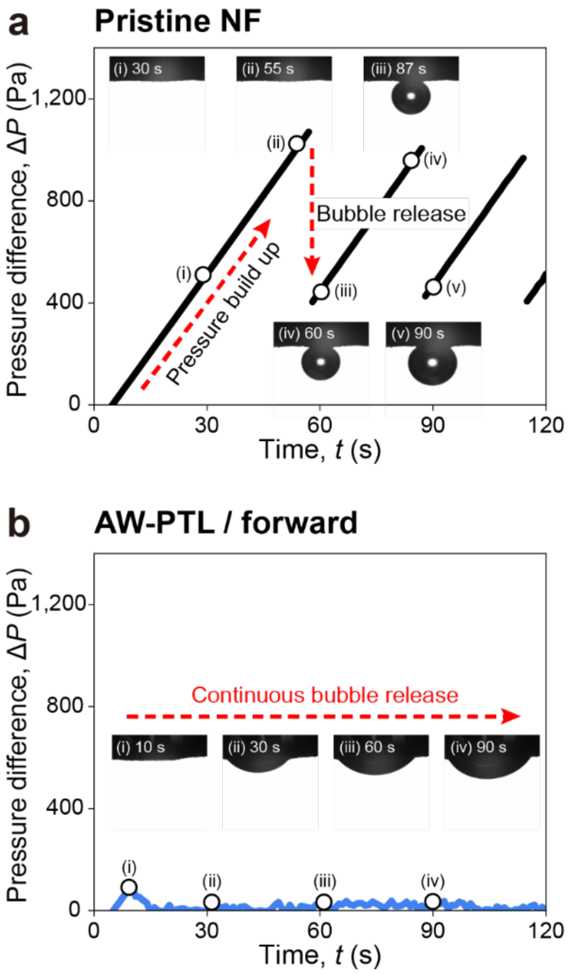

Figure S15. Pressure profiles with representative points correlated to optical images during bubble point pressure measurements for a) pristine NF and b) AW-PTL in the forward direction, highlighting the stick-slip-like cycle of pressure build-up and bubble release.

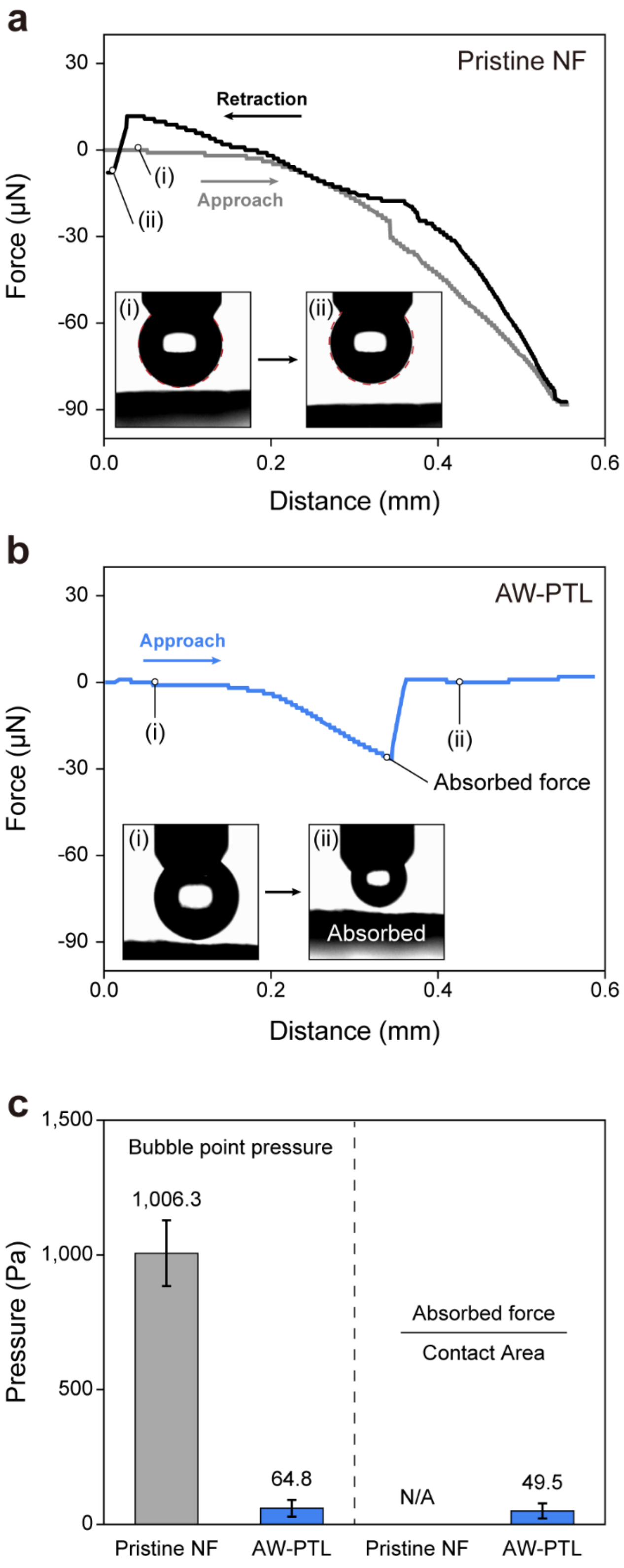

Figure S16. Bubble-absorbing force measurement. a, b) Force-distance profiles of water droplets on pristine NF and AW-PTL in the forward direction. c) Comparison of BPPs with calculated bubble-absorbing pressure derived from the force-distance profiles.

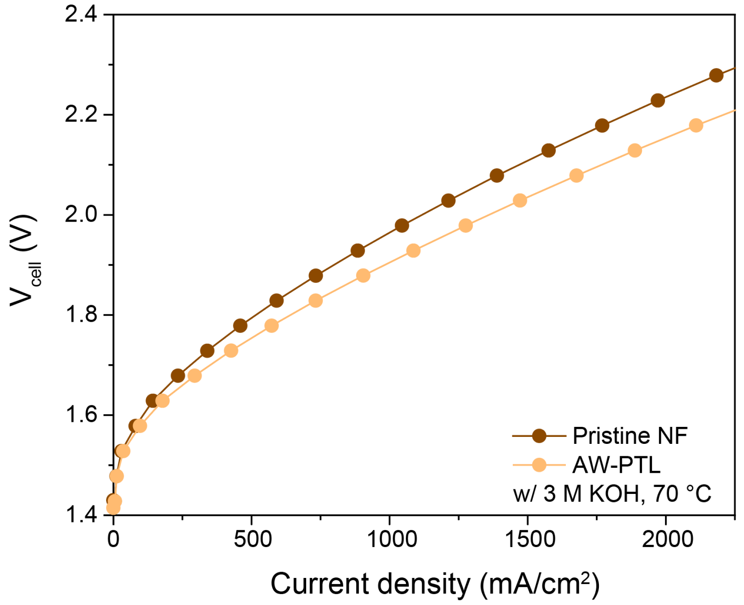

Figure S17. I-V characteristics of AEMWEs with pristine NF and AW-PTL, measured in 3 M KOH at 70 °C. The results confirm that AW-PTLs maintain improved performance even under harsher alkaline conditions.

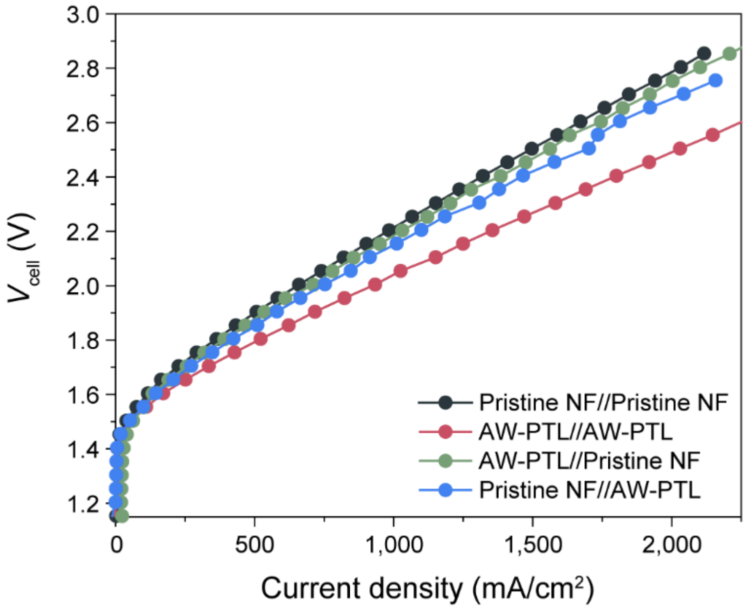

Figure S18. Effects of PTL configurations on AEMWE performance. AEMWEs were assembled using pristine NF and AW-PTL in various combinations as PTLS for the cathode and the anode.

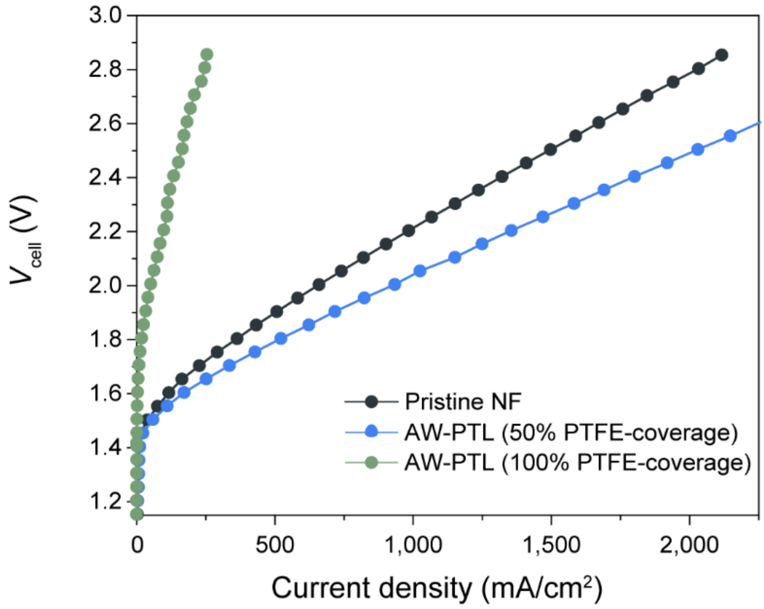

Figure S19. Effect of PTFE coverage on AEMWE performance with AW-PTLs. AW-PTL with 50% PTFE-coated area on one side shows optimized performance.

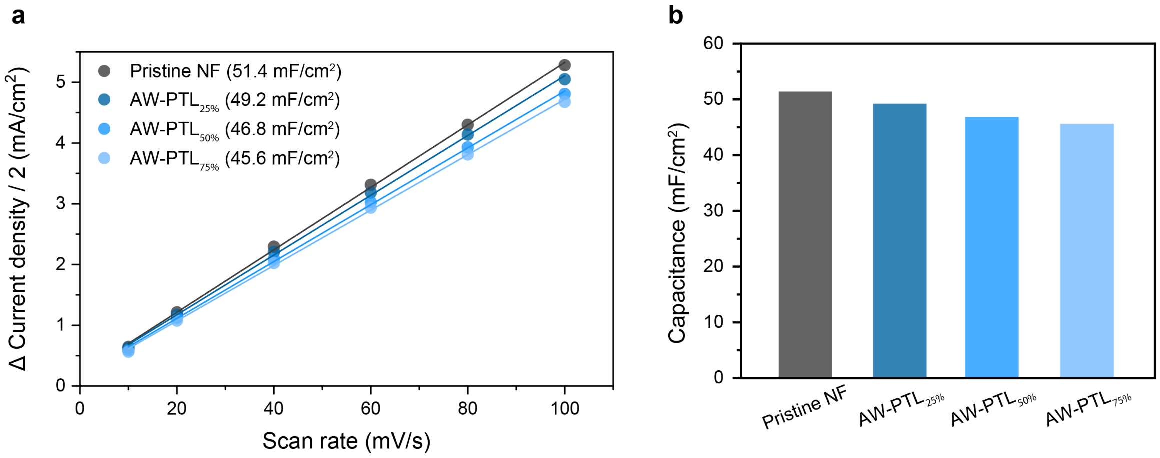

Figure S20. Evaluation of electrochemical double-layer capacitance to estimate the ECSA of pristine NF and AW-PTLs. a) Linear relationship between current density and scan rate, used to extract capacitive values. b) Comparison of capacitances for different PTFE coverage ratios (25%, 50%, 75%), showing a slight decrease in ECSA with increasing hydrophobic surface area. For AW-PTL$_{50\%}$, the measured ECSA exhibited only a ~9 % decrease after PTFE modification compared with pristine NF.

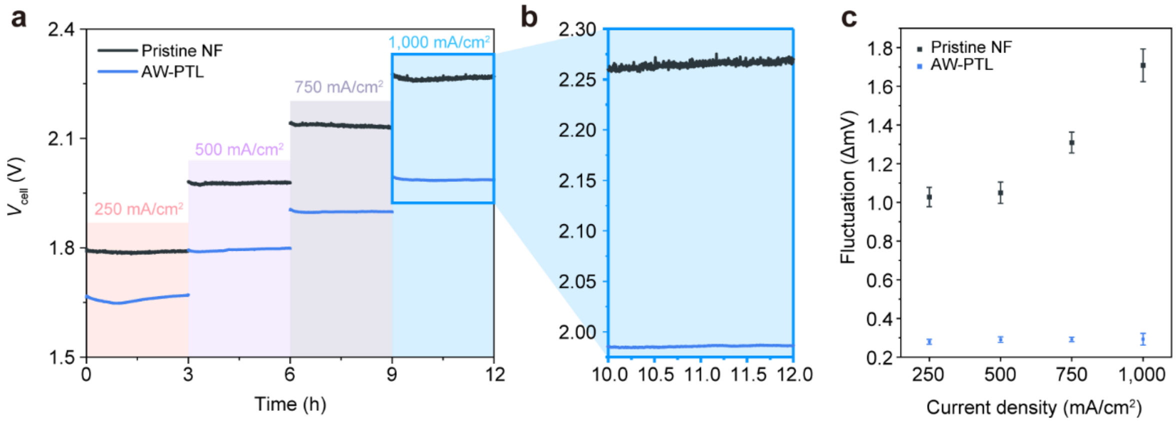

Figure S21. Stepwise chronopotentiometry tests. a, b) I-t curves of AEMWEs employing pristine NF and AW-PTL at each current density, with an enlarged graph at 1000 mA/cm^2. c) Voltage fluctuations under various conditions, showing a marked suppression of fluctuations in AW-PTL.

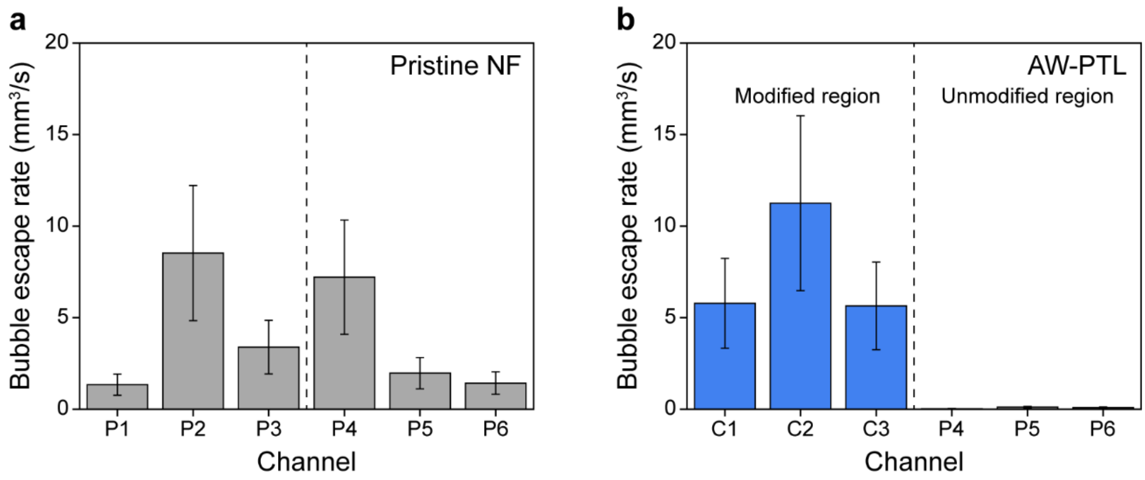

Figure S22. Comparison of bubble escape rate. a, b) Bubble escape rates in the channels of AEMWE cells using a) pristine NF and b) AW-PTL.

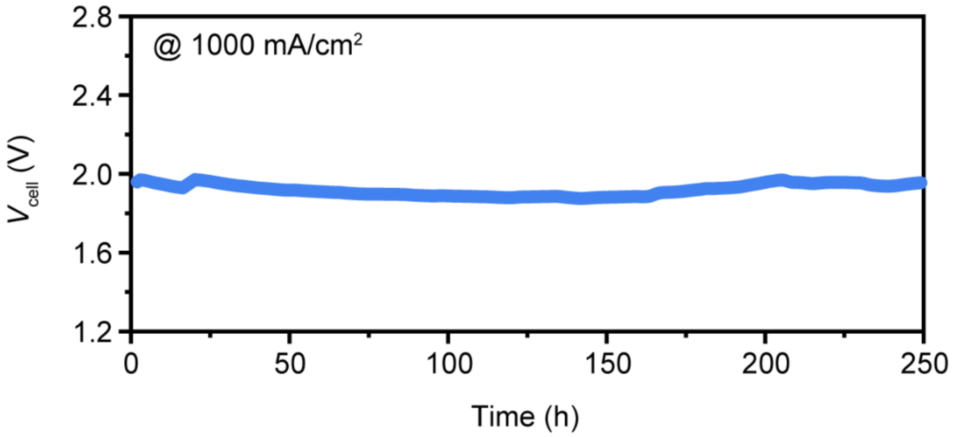

Figure S23. Chronoamperometric durability test of an AW-PTL operated at a current density of 1000 mA/cm^2 for 250 h. The cell voltage remained highly stable near 2.0 V throughout the test, demonstrating the excellent long-term durability of the AW-PTL under industrially relevant current densities.

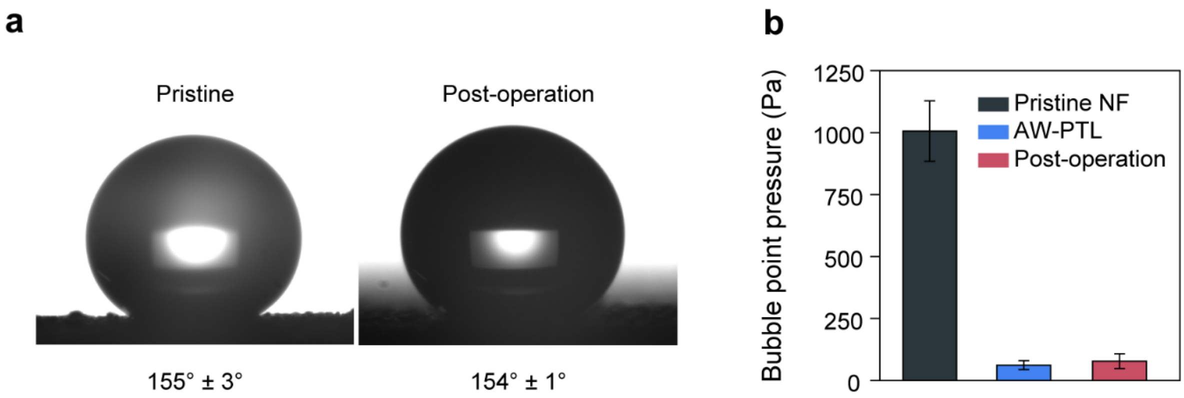

Figure S24. Durability of AW-PTLs. a) Maintenance of superhydrophobic properties after 150 h of water electrolysis. b) Comparison of BPPs of AW-PTL before and after water electrolysis.

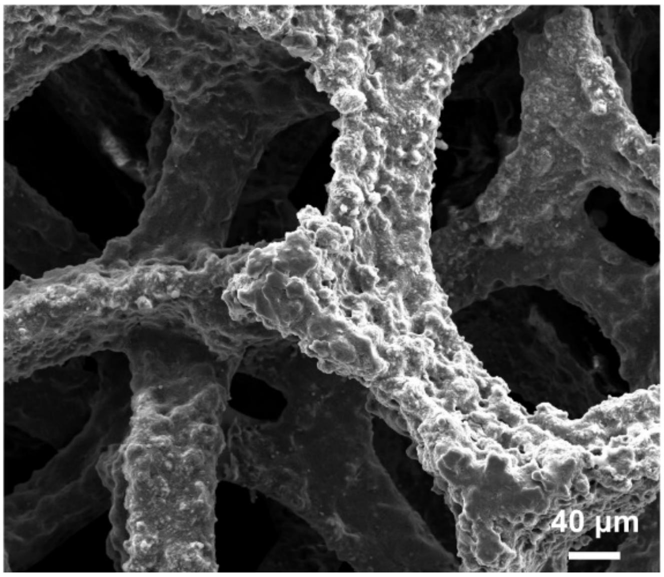

Figure S25. Morphology of AW-PTL post-operation. After chronopotentiometry tests at 500 mA/cm^2 for 150 h, the PTFE was found to be stably retained on Ni framework.

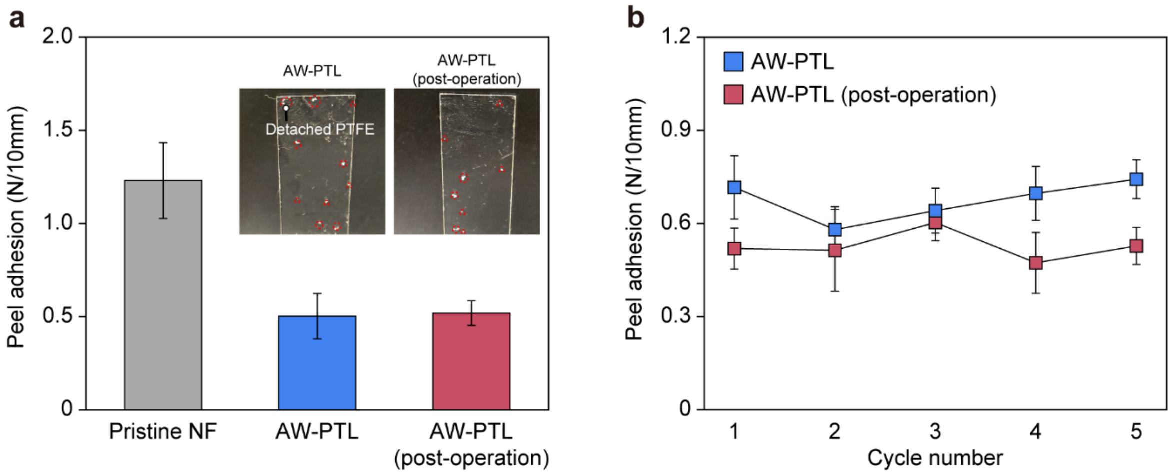

Figure S26. Stability of PTFE layers in AW-PTLs. a) Peel strength of PTLs measured using 3M double-coated tape. (inset) Images of the tape after the peel test, showing that only a few PTFE fragments were detached while most of the PTFE layer remained intact b) Peel strength of AW-PTL and AW-PTL (post-operation) over five attach–peel cycles.

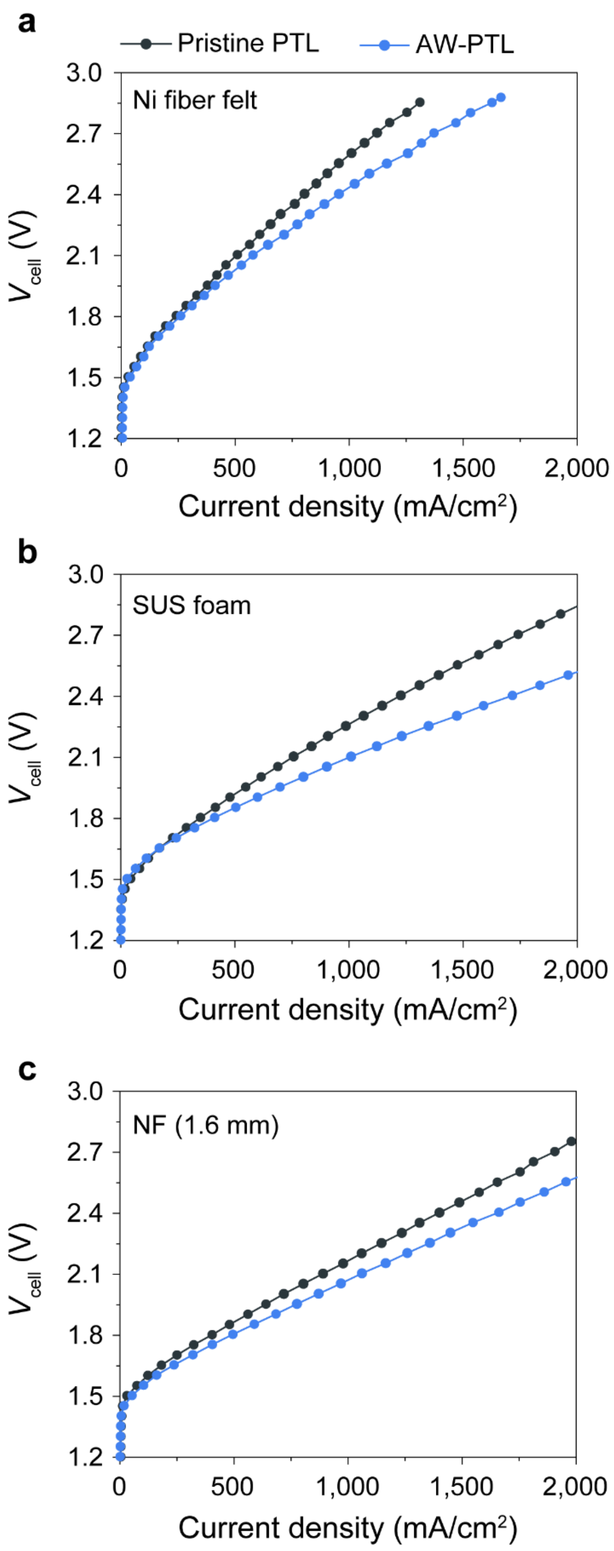

Figure S27. Performance of various PTLs in AEMWEs. (a-c) I-V curves of various PTLs before and after modification into AW-PTLs: a) Ni felt, b) SUS foam, and c) and NF with 1.6 mm thickness.

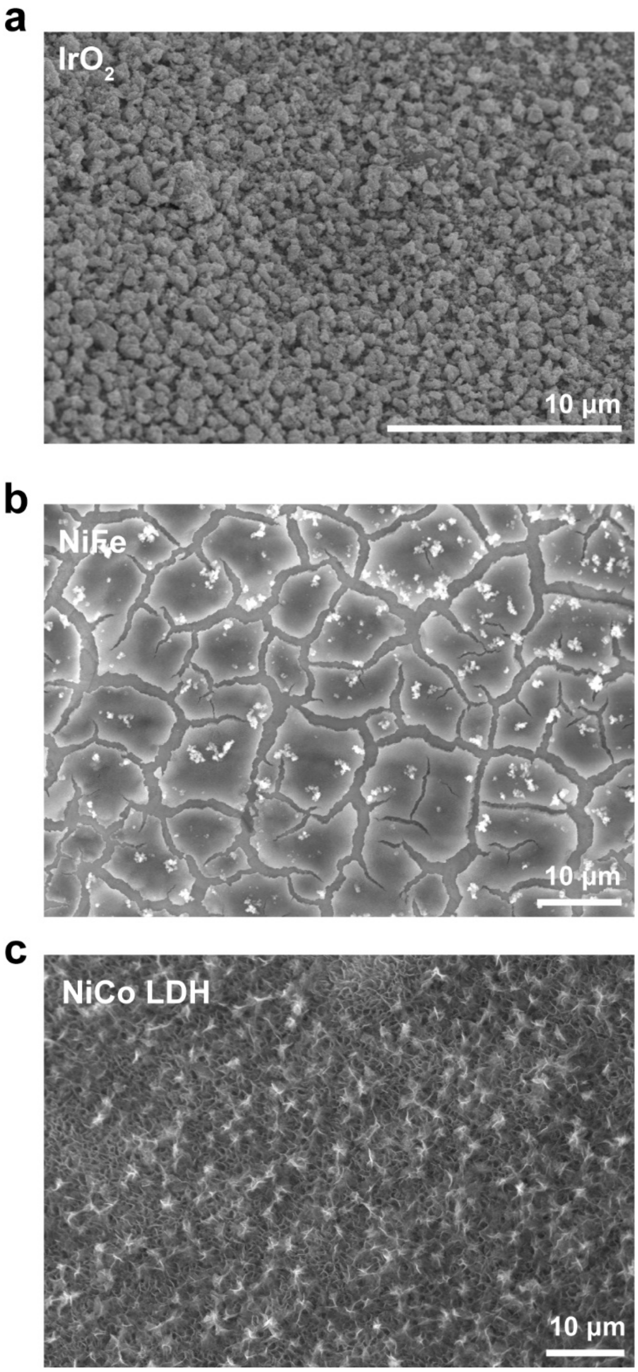

Figure S28. Morphology of various OER electrocatalysts tested in this study. a-c) Electron micrographs of a) IrO_2, b) NiFe, and c) NiCo LDH.

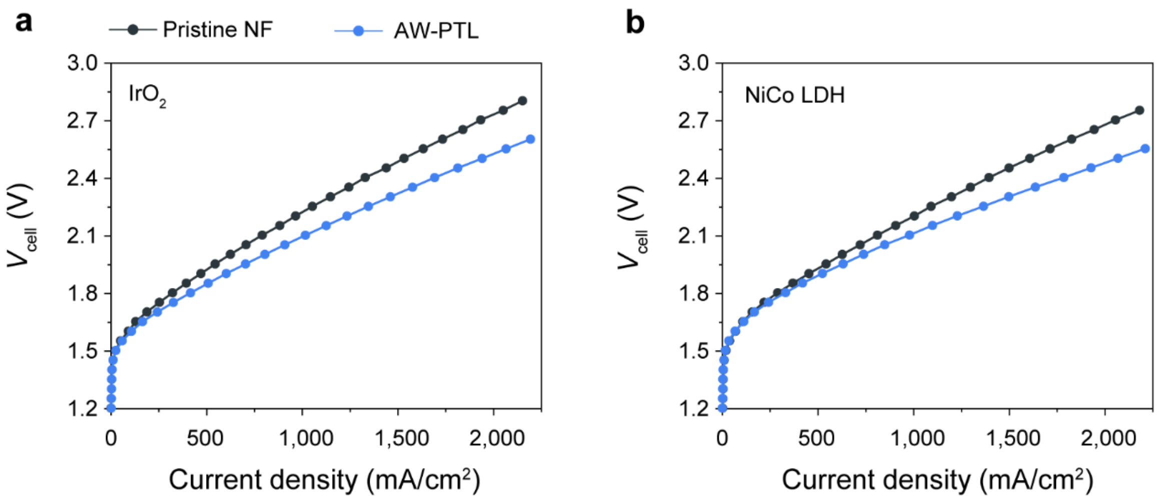

Figure S29. Combination of NF-based AW-PTL with various OER catalysts. a, b) Evaluation of AW-PTL on the electrochemical performances of various electrocatalysts: a) IrO_2 and b) NiCo LDH.

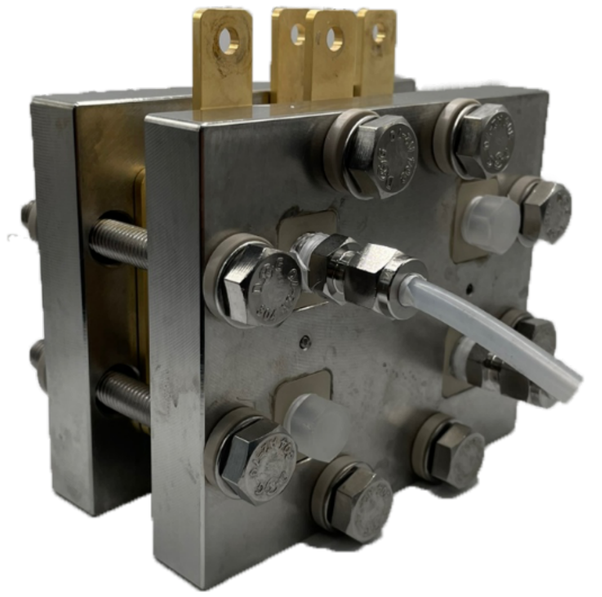

Figure S30. stack electrolyzer tested in this study. Photograph of the 3-stack cell assembly with Au-coated current collector.

Table S1. Comparison of different strategies for controlling wettability in various components of water electrolyzers.

Catalyst	Support	Method	Target reaction	Area	Feed	Stability	Ref.
IrO_2, Pt, NiCo, NiFe	Ni, SUS	Spray coating for PTL modification	HER OER	225 cm^2 MEA	1 M KOH	250 h at 1000 mA/cm^2 using a 25 cm^2 MEA	This work
Pt	Ti	Lithographic patterning of electrodes	HER	2.25 cm^2	0.5 M H_2SO_4	30 h at 500 mA/cm^2	[S1]
IrO_2, Pt	Ti felt	Shadow mask patterning for PTL modification	OER ORR	2.25 cm^2 MEA	0.1 M $HClO_4$	160 h at 500 mA/cm^2	[S2]
Ir, Pt	Ni	Air plasma spraying for deposition of microporous layers on PTL	HER OER	4 cm^2 MEA	1 M KOH	170 h at 500 mA/cm^2	[S3]
$Fe_{0.8}Ni_{0.2}P_{0.5}S_{0.5}$	Ni	Laser etching and electrodeposition for electrode modification	HER OER	2.25 cm^2	1 M KOH	35 h at -0.062 V_{RHE} for HER / 1.33 V_{RHE} for OER	[S4]

Table S2. Wettability of pristine NF and AW-PTL.

Unit [°]	Water contact angle	Air contact angle	Water sliding angle	Water advancing angle	Water receding angle
Pristine NF	N/A	145.7	N/A	31.5	30.8
Ni side (AW-PTL)	N/A	N/A	N/A	30.2	24.8
PTFE side (AW-PTL)	154.8	8.9	7.6	160.6	144.6

Table S3. Comparison of zero-gap electrolyzer performances under similar operating conditions.

Catalysts	System	Electrolyte	Performance	Reference
NiFe, Pt/C	Zero-gap water electrolyzer with AW-PTL	1 M KOH	2.1 V at 1151 mA/cm^2	This work
	Zero-gap water electrolyzer with pristine PTL	1 M KOH	2.1 V at 820 mA/cm^2	
Ni, Pt/C	Zero-gap water electrolyzer	1 M KOH	2.1 V at 1000 mA/cm^2	[S5]
IrO_2, $NiMoO_4$	Zero-gap water electrolyzer	1 M KOH	2.2 V at 1000 mA/cm^2	[S6]
$La_{0.8}Sr_{0.2}CoO_3$, $Fe_4Co_3Ni_2S_8$	Zero-gap water electrolyzer	1 M KOH	2.22 V at 1000 mA/cm^2	[S7]
$NiCoO_x$, Pt/C	Zero-gap water electrolyzer	1 M KOH	2.1 V at 1000 mA/cm^2	[S8]

Supporting References

[S1] *Sci. Adv.* **2023**, *9*, eadd6978.

[S2] *Sci. Adv.* **2021**, *7*, eabf7866.

[S3] *Joule* **2021**, *5*, 1776.

[S4] *Adv. Mater*. **2024**, 36, 2405493.

[S5] *Mater. Renew. Sustain. Energy* **2025**, 14, 47

[S6] *Int. J. Hydrogen Energy* **2025**, 104, 416

[S7] *Adv. Energy Sustain. Res*. **2024**, 5, 2400128

[S8] *J. Energy Storage* **2024**, 79, 110149
